# Machine learning‐based prediction of clinical outcomes after traumatic brain injury: Hidden information of early physiological time series

**DOI:** 10.1111/cns.14848

**Published:** 2024-07-07

**Authors:** Ruifeng Ding, Mengqiu Deng, Huawei Wei, Yixuan Zhang, Liangtian Wei, Guowei Jiang, Hongwei Zhu, Xingshuai Huang, Hailong Fu, Shuang Zhao, Hongbin Yuan

**Affiliations:** ^1^ Department of Anesthesiology, Changzheng Hospital Second Affiliated Hospital of Naval Medical University Shanghai China; ^2^ Jiangsu Province Key Laboratory of Anesthesiology Xuzhou Medical University Xuzhou China; ^3^ Department of Anesthesiology The Third Hospital of Hebei Medical University Shijiazhuang Hebei Province China

**Keywords:** electronic health record, interpretable algorithms, physiological time series, prognostic features, traumatic brain injury

## Abstract

**Aims:**

To assess the predictive value of early‐stage physiological time‐series (PTS) data and non‐interrogative electronic health record (EHR) signals, collected within 24 h of ICU admission, for traumatic brain injury (TBI) patient outcomes.

**Methods:**

Using data from TBI patients in the multi‐center eICU database, we focused on in‐hospital mortality, neurological status based on the Glasgow Coma Score (mGCS) motor subscore at discharge, and prolonged ICU stay (PLOS). Three machine learning (ML) models were developed, utilizing EHR features, PTS signals collected 24 h after ICU admission, and their combination. External validation was performed using the MIMIC III dataset, and interpretability was enhanced using the Shapley Additive Explanations (SHAP) algorithm.

**Results:**

The analysis included 1085 TBI patients. Compared to individual models and existing scoring systems, the combination of EHR and PTS features demonstrated comparable or even superior performance in predicting in‐hospital mortality (AUROC = 0.878), neurological outcomes (AUROC = 0.877), and PLOS (AUROC = 0.835). The model's performance was validated in the MIMIC III dataset, and SHAP algorithms identified six key intervention points for EHR features related to prognostic outcomes. Moreover, the EHR results (All AUROC >0.8) were translated into online tools for clinical use.

**Conclusion:**

Our study highlights the importance of early‐stage PTS signals in predicting TBI patient outcomes. The integration of interpretable algorithms and simplified prediction tools can support treatment decision‐making, contributing to the development of accurate prediction models and timely clinical intervention.

## Graphical abstract

A novel method predicts outcomes in traumatic brain injury (TBI) patients using electronic health record (EHR) data and physiological time‐series (PTS) signals. Interpretable machine learning techniques identify effective models and offer clinical insights.

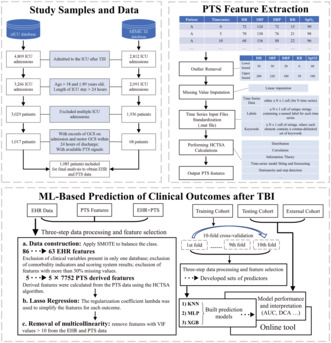

## 1 INTRODUCTION

Traumatic brain injury (TBI) encompasses cranial injuries caused by traffic accidents, falls, sports, and war. Globally, TBI accounts for approximately 69 million cases each year, constituting a significant cause of death and disability. About 10% of TBI cases necessitate intensive care unit (ICU) admission.^1^, ^2^

Identifying early predictors of patient prognosis and outcomes, as well as implementing effective treatments, poses major challenges in TBI research.^3^ The APACHE score is commonly utilized to forecast the severity and prognosis of critically ill patients.^4^ However, obtaining timely indicators remains a challenging endeavor. For instance, TBI patients admitted to the emergency department often present in a state of coma, impeding the timely collection of detailed information regarding chronic comorbidities.^5^ Moreover, gathering over 100 variables required for calculating the APACHE score poses additional difficulties due to limited medical resources available.

In recent years, machine learning (ML) has gained significant attention and recognition in the clinical field due to advancements in statistical theory and computer technology. However, most studies only incorporate repeated measurements of the same variable at different time points (longitudinal data). A study of publications from 2009 to 2016 revealed that less than 8% of predictive models included longitudinal data as time‐varying covariates.^6^ Neglecting the potential of these high‐dimensional, longitudinal, time‐varying covariates to improve prediction, as demonstrated in cardiovascular disease risk prediction,^7^, ^8^ is a prevailing issue.

In this study, we propose a novel method for predicting clinical outcomes of TBI patients, including survival, neurological function, and prolonged length of ICU stay (PLOS), based on clinical electronic health record (EHR) data and physiological time‐series (PTS) signals. Additionally, we employ interpretable ML techniques to elucidate the best‐performing models and gain valuable clinical insights. The findings are validated using comprehensive data from the multi‐center Philips eICU‐CRD database^9^ and additionally validated using the Medical Information Mart for Intensive Care (MIMIC) III database.^10^

## 2 MATERIALS AND METHODS

### 2.1 Study design and protocol

The study design was implemented as depicted in graphical abstract image. The author of this study has successfully completed the Collaborative Institutional Training Initiative examination (certification number 49437998) and was granted access to both databases. The author was responsible for extracting the data. Given that the study involved the analysis of anonymized publicly available databases, which had obtained prior institutional review board approval from the Massachusetts Institute of Technology and BIDMC, the requirement for informed consent was waived.

### 2.2 Study samples

All patients included in this study were sourced from the eICU version v2.0 databases and MIMIC‐III version v1.4. Patients with a diagnosis of TBI were identified in both databases using International Classification of Diseases‐9 diagnosis codes (Table S6). The study employed the following inclusion criteria: (1) admission to the ICU following TBI; (2) age between 18 and 89 years; (3) ICU stay duration exceeding 24 h; (4) availability of PTS signals; and (5) presence of Glasgow Coma Scale (GCS) records at admission and motor GCS records within 24 h of discharge. In cases where patients had multiple ICU admissions, only data from the initial ICU admission during the first hospitalization were considered for analysis. One point that needs to be mentioned is that PTS signals are not currently available in MIMIC‐IV; hence, they cannot be obtained. Therefore, MIMIC‐III database was used for external validation, despite it being released earlier.

### 2.3 Variable extraction

Clinical variables from the initial 24 h of ICU stay for TBI patients were extracted from both databases using structured query language for further analysis. The EHR data collected encompassed various domains, including demographics, comorbidities, laboratory results, vital signs, medical treatments, and scoring systems. An overview of the selected EHR features is provided in Table S7.

PTS data in the eICU database were recorded at 5‐min intervals and included heart rate (HR), systolic and diastolic blood pressure (SBP, DBP), respiratory rate, and oxygen saturation measured by pulse oximeter (SpO2). Similarly, PTS data for patients in the MIMIC‐III database were extracted from the MIMIC‐III waveform database^11^ based on matching identification. To achieve the objectives of our study, we specifically focused on the PTS data obtained within the initial 24 h following ICU admission for the aforementioned five signals. Each patient's PTS data were stored individually in a separate file to facilitate subsequent feature extraction analysis.

### 2.4 Outcome definition

This study aimed to investigate three primary clinical outcomes: in‐hospital mortality, neurological status at hospital discharge, and PLOS. Neurological outcome was assessed using the motor subscore of the Glasgow Coma Score (mGCS) recorded at discharge, where a favorable outcome was defined as an mGCS score of 6 and an unfavorable outcome as an mGCS score ≤5. PLOS was considered as a binary variable, categorized based on the 75th percentile length of ICU stay among the study participants. In the eICU cohort, a PLOS of 5 days or longer was classified as prolonged, while in the MIMIC III cohort, a PLOS of 16 days or longer was considered prolonged.

## 3 RESULTS

### 3.1 Study participants

Initially, the entire population from the two databases was screened, resulting in the identification of 4809 ICU admissions after TBI from the eICU‐CRD database and 2812 admissions from the MIMIC‐III database, respectively. Following the application of exclusion criteria, a total of 1085 patients were ultimately included in the study cohort. Detailed summaries of the characteristics and outcome distributions of the TBI cohorts are provided in Tables 1 and S8, respectively.

**TABLE 1:** Demographic and clinical characteristics of the complete datasets.

	eICU‐CRD	MIMIC III	*p*‐Value
*n*	1017	68	
Demographic			
Age	58.33 (20.82)	61.00 (21.64)	0.327
Male	453 (44.54%)	49 (72.06%)	<0.001
Race			
Asian	13 (1.28%)	2 (2.94%)	<0.001
Black	99 (9.73%)	4 (5.88%)	
Hispanic	27 (2.65%)	2 (2.94%)	
Other/Unknown	67 (6.59%)	18 (26.47%)	
Weight	81.93 (21.34)	81.73 (16.66)	0.924
Intracranial injury types			
Epidural hematoma	42 (4.13%)	16 (23.53%)	<0.001
Subdural hematoma	434 (42.67%)	16 (23.53%)	0.003
Subarachnoid hemorrhage	347 (34.12%)	16 (23.53%)	0.097
Intracerebral hemorrhage	187 (17.16%)	0 (0.00%)	<0.001
Cerebral contusion	57 (5.60%)	13 (19.12%)	<0.001
Severity scores on admission			
GCS	10.98 (4.61)	12.04 (3.98)	0.038
APSIII	47.80 (27.08)	46.90 (24.34)	0.771
SOFA	3.80 (3.01)	4.19 (2.88)	0.286
First day treatment			
Vasopressor	68 (6.69%)	26 (38.24%)	<0.001
Renal replacement therapy	9 (0.88%)	1 (1.47%)	0.478
Mechanical ventilation	438 (43.07%)	47 (69.12%)	<0.001
Neurosurgery	80 (7.87%)	31 (45.59%)	<0.001
Hospital length of stay, day	8.56 (9.77)	8.50 (9.07)	0.959
ICU length of stay, day	5.25 (6.34)	7.99 (8.39)	0.010
Outcomes			
In‐hospital mortality			
Alive	825 (81.12%)	39 (57.35%)	<0.001
Expired	185 (18.19%)	29 (42.65%)	
Neurological outcome			
Favorable	739 (72.66%)	33 (48.53%)	<0.001
Unfavorable	278 (27.34%)	35 (51.47%)	
Prolonged length of ICU stay			
NO	607 (73.58%)	29 (74.36%)	1.000
YES	218 (26.42%)	10 (25.64%)	

*Note*: Data are *n* (%) or mean (SD).

Abbreviations: APSIII, Acute Physiology Score III; GCS, Glasgow Coma Scale; SOFA, Sequential Organ Failure Assessment.

### 3.2 Variables selected for each clinical outcome

Figures S1 and S2 illustrate the feature variable selection results of LASSO regression applied to identify the EHR data and PTS data for each clinical outcome. After conducting a multicollinearity check, it was ensured that the VIF values of the included indicators were all less than 10, as shown in Figure S3. Ultimately, a total of 32 clinical indicators and 8 PTS variables were selected for predicting in‐hospital mortality. For neurological outcome, 29 clinical indicators and 23 PTS variables were chosen. Lastly, for PLOS, 13 clinical indicators and 11 PTS variables were selected.

### 3.3 Model performance

Table 2, Figures S4–S7, along with Tables S8 and S9, present the performance of all predictive models for each clinical outcome. The evaluation encompassed different feature subsets, including EHR‐only, PTS‐only, and the combined EHR and PTS, as well as various scoring systems such as APACHE, APS, GCS, and SOFA.

**TABLE 2:** Model performance summary for all models for each clinical outcome and feature subset in testing set.

	Source	Model type	AUROC	Sensitivity	Specificity	F1 score	Accuracy
In‐hospital mortality	EHR	KNN	0.691 (0.615, 0.766)	0.500 (0.375, 0.625)	0.794 (0.741, 0.842)	0.415 (0.360, 0.472)	0.739 (0.690, 0.789)
		MLP	0.713 (0.640, 0.786)	0.786 (0.679, 0.893)	0.543 (0.482, 0.603)	0.413 (0.356, 0.469)	0.587 (0.531, 0.644)
		*XGBoost	0.869 (0.819, 0.919)	0.839 (0.732, 0.929)	0.789 (0.737, 0.838)	0.606 (0.551, 0.660)	0.799 (0.752, 0.842)
	PTS	KNN	0.740 (0.673, 0.807)	0.536 (0.411, 0.661)	0.818 (0.769, 0.866)	0.458 (0.403, 0.515)	0.766 (0.716, 0.812)
		*MLP	0.816 (0.752, 0.880)	0.732 (0.607, 0.839)	0.806 (0.757, 0.854)	0.566 (0.508, 0.620)	0.792 (0.746, 0.838)
		XGBoost	0.813 (0.751, 0.875)	0.839 (0.732, 0.929)	0.680 (0.619, 0.737)	0.516 (0.459, 0.574)	0.710 (0.657, 0.759)
	EHR + PTS	KNN	0.725 (0.644, 0.805)	0.589 (0.464, 0.714)	0.822 (0.773, 0.866)	0.496 (0.439, 0.551)	0.779 (0.733, 0.825)
		MLP	0.826 (0.768, 0.884)	0.750 (0.625, 0.857)	0.810 (0.761, 0.858)	0.579 (0.525, 0.634)	0.799 (0.752, 0.842)
		*XGBoost	0.878 (0.832, 0.925)	0.911 (0.821, 0.982)	0.741 (0.684, 0.794)	0.596 (0.541, 0.650)	0.772 (0.723, 0.818)
	APACHE IV		0.908 (0.865, 0.950)	0.889 (0.796, 0.963)	0.784 (0.730, 0.838)	0.640 (0.583, 0.696)	0.804 (0.757, 0.851)
	APS III		0.895 (0.845, 0.945)	0.815 (0.704, 0.907)	0.842 (0.793, 0.887)	0.662 (0.605, 0.717)	0.837 (0.793, 0.880)
	GCS		0.805 (0.739, 0.872)	0.732 (0.607, 0.839)	0.797 (0.743, 0.846)	0.562 (0.505, 0.616)	0.785 (0.737, 0.832)
	SOFA		0.859 (0.812, 0.906)	0.821 (0.714, 0.911)	0.749 (0.692, 0.802)	0.561 (0.505, 0.617)	0.762 (0.713, 0.809)
Neurological Status	EHR	KNN	0.681 (0.613, 0.749)	0.512 (0.405, 0.619)	0.770 (0.712, 0.824)	0.483 (0.428, 0.539)	0.699 (0.647, 0.752)
		MLP	0.733 (0.667, 0.800)	0.607 (0.500, 0.714)	0.784 (0.730, 0.838)	0.557 (0.500, 0.614)	0.735 (0.686, 0.784)
		*XGBoost	0.863 (0.817, 0.908)	0.786 (0.690, 0.869)	0.842 (0.793, 0.887)	0.714 (0.663, 0.765)	0.827 (0.784, 0.869)
	PTS	KNN	0.734 (0.669, 0.798)	0.583 (0.476, 0.690)	0.797 (0.743, 0.847)	0.551 (0.493, 0.605)	0.739 (0.690, 0.788)
		MLP	0.659 (0.596, 0.721)	0.869 (0.798, 0.940)	0.419 (0.356, 0.482)	0.510 (0.454, 0.565)	0.542 (0.487, 0.598)
		*XGBoost	0.805 (0.751, 0.858)	0.810 (0.726, 0.893)	0.689 (0.626, 0.748)	0.615 (0.562, 0.670)	0.722 (0.670, 0.771)
	EHR + PTS	KNN	0.753 (0.691, 0.814)	0.619 (0.512, 0.726)	0.806 (0.752, 0.856)	0.581 (0.526, 0.637)	0.755 (0.706, 0.801)
		MLP	0.777 (0.715, 0.839)	0.655 (0.548, 0.750)	0.847 (0.797, 0.892)	0.636 (0.582, 0.690)	0.794 (0.748, 0.840)
		*XGBoost	0.877 (0.833, 0.921)	0.845 (0.762, 0.917)	0.766 (0.707, 0.820)	0.686 (0.634, 0.739)	0.788 (0.742, 0.833)
	APACHE IV		0.845 (0.798, 0.892)	0.908 (0.839, 0.966)	0.624 (0.558, 0.690)	0.658 (0.602, 0.711)	0.711 (0.658, 0.764)
	APS III		0.836 (0.786, 0.885)	0.874 (0.805, 0.943)	0.675 (0.609, 0.741)	0.670 (0.613, 0.725)	0.736 (0.683, 0.785)
	GCS		0.776 (0.718, 0.834)	0.671 (0.573, 0.768)	0.817 (0.766, 0.867)	0.621 (0.567, 0.677)	0.777 (0.730, 0.823)
	SOFA		0.789 (0.735, 0.843)	0.780 (0.692, 0.857)	0.679 (0.614, 0.740)	0.615 (0.559, 0.670)	0.709 (0.657, 0.758)
Prolonged length of ICU stay	EHR	KNN	0.641 (0.568, 0.713)	0.879 (0.788, 0.955)	0.390 (0.319, 0.462)	0.494 (0.431, 0.556)	0.520 (0.460, 0.581)
		MLP	0.662 (0.580, 0.744)	0.652 (0.530, 0.758)	0.665 (0.593, 0.731)	0.506 (0.444, 0.569)	0.661 (0.601, 0.718)
		*XGBoost	0.803 (0.744, 0.863)	0.909 (0.833, 0.970)	0.593 (0.522, 0.665)	0.600 (0.540, 0.661)	0.677 (0.617, 0.734)
	PTS	KNN	0.790 (0.723, 0.856)	0.758 (0.652, 0.864)	0.769 (0.709, 0.830)	0.543 (0.573, 0.694)	0.766 (0.714, 0.819)
		* MLP	0.821 (0.761, 0.881)	0.636 (0.515, 0.742)	0.879 (0.830, 0.923)	0.646 (0.585, 0.706)	0.815 (0.766, 0.863)
		XGBoost	0.739 (0.670, 0.809)	0.742 (0.636, 0.848)	0.648 (0.577, 0.714)	0.547 (0.484, 0.609)	0.673 (0.613, 0.730)
	EHR + PTS	KNN	0.642 (0.570, 0.714)	0.682 (0.561, 0.788)	0.582 (0.511, 0.654)	0.481 (0.419, 0.544)	0.609 (0.548, 0.669)
		MLP	0.825 (0.768, 0.882)	0.773 (0.667, 0.864)	0.808 (0.747, 0.863)	0.671 (0.613, 0.730)	0.798 (0.746, 0.847)
		*XGBoost	0.835 (0.781, 0.890)	0.773 (0.667, 0.864)	0.775 (0.714, 0.835)	0.646 (0.585, 0.706)	0.774 (0.722, 0.827)
	APACHE IV		0.696 (0.615, 0.776)	0.571 (0.446, 0.696)	0.725 (0.655, 0.789)	0.474 (0.410, 0.537)	0.687 (0.626, 0.744)
	APS III		0.711 (0.629, 0.792)	0.625 (0.500, 0.750)	0.749 (0.684, 0.813)	0.522 (0.458, 0.586)	0.718 (0.661, 0.775)
	GCS		0.778 (0.712, 0.844)	0.672 (0.562, 0.781)	0.803 (0.742, 0.860)	0.606 (0.545, 0.665)	0.769 (0.715, 0.822)
	SOFA		0.728 (0.658, 0.799)	0.803 (0.697, 0.894)	0.593 (0.522, 0.665)	0.549 (0.488, 0.613)	0.649 (0.589, 0.710)

Abbreviations: APACHE IV, Acute Physiology and Chronic Health Evaluation IV; APSIII, Acute Physiology Score III; AUROC, area under the receiver operating characteristic curve; EHR, electronic health record; GCS, Glasgow Coma Scale; KNN, K‐nearest neighbor; MLP, multi‐layer perceptron; PTS, physiological time series; SOFA, Sequential Organ Failure Assessment; XGBoost, eXtreme Gradient Boosting.

* Represents the best AUROC model in the corresponding source.

From the summary of the testing set results, we obtained the following key findings. Our results demonstrate that the individual EHR data achieved optimal AUC values greater than 0.8 for all three modeling tasks, specifically 0.869, 0.863, and 0.803, respectively. An intriguing finding is that the combination of EHR and PTS data enhances the predictive performance of all ML models, with the highest increase in AUROC being 16.3% compared to using EHR data alone. Among the three ML models, the XGBoost model demonstrates the highest discriminative ability, with AUROCs of 0.878, 0.877, and 0.835 for predicting in‐hospital mortality, neurological outcome, and PLOS, respectively. Furthermore, the decision curve analysis (DCA) indicates that the XGBoost model achieves optimal net benefit.

In order to thoroughly assess the predictive performance of our models, we conducted a comprehensive comparison with several existing scoring systems. An interesting observation is that using EHR data alone has already achieved comparable, or even surpassing, performance compared to existing scoring systems for neurological outcome and PLOS. In predicting in‐hospital mortality, the APACHE scoring system demonstrates impressive predictive performance with the highest AUROC (0.908). Although our optimized model experiences a slight decrease of approximately 3% in AUROC, it still outperforms APACHE in terms of sensitivity (0.911 vs. 0.889). Considering the in‐hospital mortality rate of 18.2%, our model appears to better avoid false negatives and has the potential to improve the identification of high‐risk patients. Given the differences in the number of metrics used in actual calculations, we consider this performance decrease acceptable. Furthermore, our optimized model exhibits higher performance in predicting neurological outcome and PLOS compared to the scoring systems. Specifically, for neurological outcome, our model achieves a 3.2% increase in AUROC compared to the scoring systems. Regarding PLOS, our model shows a notable improvement with a 5.7% increase in AUROC. Additionally, our model demonstrates significant practical utility in PLOS outcomes, with the highest PPV of 0.554 and NPV of 0.904. The Youden index can be indirectly calculated using sensitivity and specificity. Consistent with the previous results, for in‐hospital mortality, the combination of EHR and PTS features produced slightly lower but comparable performance to the existing scoring systems, with values of 0.652 vs. 0.673. However, for neurological status at discharge and PLOS, our model significantly outperformed the traditional scoring systems, with values of 0.611 vs. 0.532 and 0.548 vs. 0.296, respectively. Finally, through our external validation, it was demonstrated that the robustness of the XGBoost model and the significance of PTS within it were further confirmed, reinforcing our assertion that PTS data encapsulate a wealth of prognostic information.

### 3.4 Most important predictors of TBI

**FIGURE 1:**
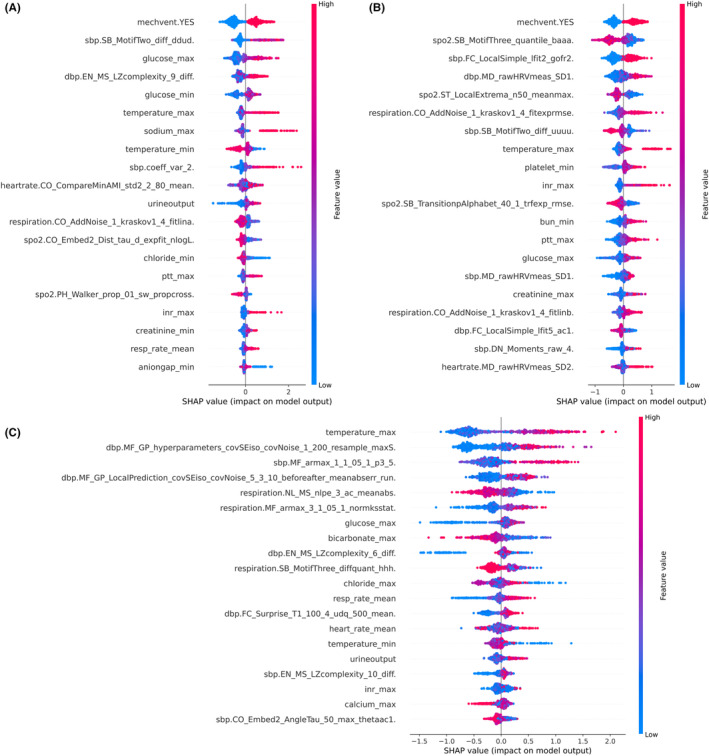
Shapley Additive Explanations analysis results of optimal prediction model XGBoost. Rank of importance of the top 20 features for in‐hospital mortality (A), neurological status at hospital discharge (B), and prolonged length of ICU stay (C).

The SHAP algorithm was conducted to visually exhibit each factor's importance to the three clinical outcomes predicted by the optimal model XGBoost. Figure 1 shows the feature importance plot, including the top 20 selected EHR and PTS variables in descending order. Briefly, a higher intensity of red indicates a greater risk impact, while a higher intensity of blue indicates a greater protective impact.

**FIGURE 2:**
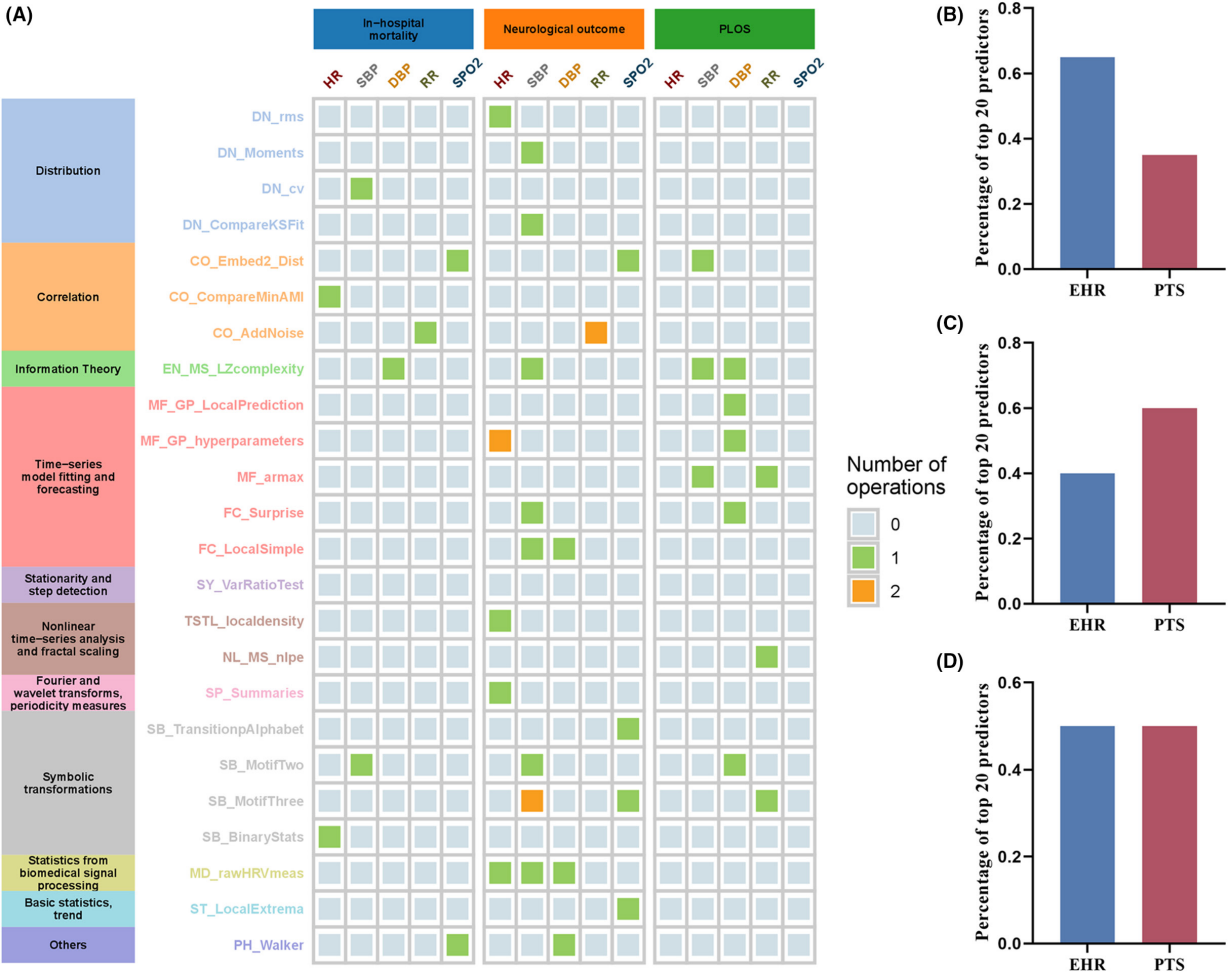
Overall plot for the highly comparative time‐series analysis physiological time‐series‐derived features. (A) Simplified distribution heatmap of highly comparative time‐series analysis physiological time‐series‐derived features for the three clinical outcomes (the complete dictionary is provided in Appendix S1). The percentage of physiological time‐series data among the top 20 variables in the prediction of outcomes in‐hospital mortality (B), neurological status at hospital discharge (C), and prolonged length of ICU stay (D), respectively, in the combined electronic health record and physiological time‐series data prediction.

In terms of in‐hospital mortality and neurological functional status, the use of mechanical ventilation appears to be the most significant factor, while the maximum temperature within 24 h of admission is associated with the state of PLOS. As depicted in Figure 2, we summarize the derived categories of PTS generated by HCTSA. In terms of all clinical outcomes, the main features contributing to in‐hospital mortality are HR, SBP, and SPO2; SBP is the primary derived feature for neurological functional status, and DBP is the primary derived feature for PLOS. Further observations reveal that PTS features account for a considerable proportion, unexpectedly reaching 60%, of the top 20 selected EHR and PTS variables for neurological functional status. This reinforces our hypothesis that these PTS features independently contribute to the final model performance.

**FIGURE 3:**
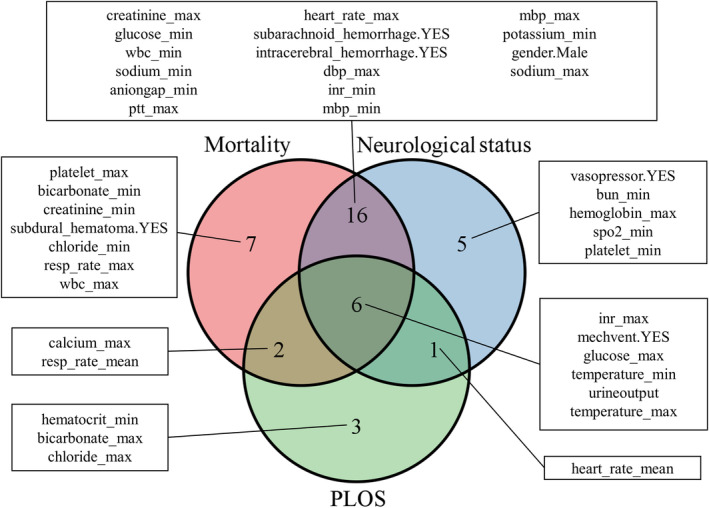
The Venn diagram summarizes the common and unique important electronic health record variables of the optimal prediction model, XGBoost, for the three clinical outcomes.

Furthermore, to obtain the precise forms of EHR factors influencing all clinical outcomes, we provide detailed descriptions using Venn diagrams and SHAP dependency plots for better clinical guidance. The analysis results are shown in Figure 3 and Figures S8 and S9. In summary, for all clinical outcomes, inr_max, mechvent, glucose_max, temperature, and urine output are considered to promote both favorable and adverse outcomes. For in‐hospital mortality, high levels of platelet_max, bicarbonate_min, creatinine_min, and subdural hematoma, as well as low levels of chloride_min, resp_rat_max, and wbc_max, are identified as individual risk factors (Figure S10). Regarding neurological function, high levels of bun_min and platelet_min, as well as low levels of hemoglobin_max, spo2_min, and the use of vasopressors, are identified as individual risk factors (Figure S11). For PLOS, high levels of hematocrit_min and chloride_max, as well as low levels of bicarbonate_max, are identified as individual risk factors (Figure S12).

### 3.5 External validation

Compared to eICU, the validation of MIMIC‐III demonstrated a decrease in performance: The AUROC of the optimal MIMIC‐III model was 0.779 for in‐hospital mortality, 0.78 for neurological prognosis, and 0.769 for PLOS, representing a decrease of 9.9%, 9.7%, and 6.6%, respectively. A comprehensive overview of the external validation results is provided in Table S10.

### 3.6 Online tool for prediction of clinical outcomes

**FIGURE 4:**
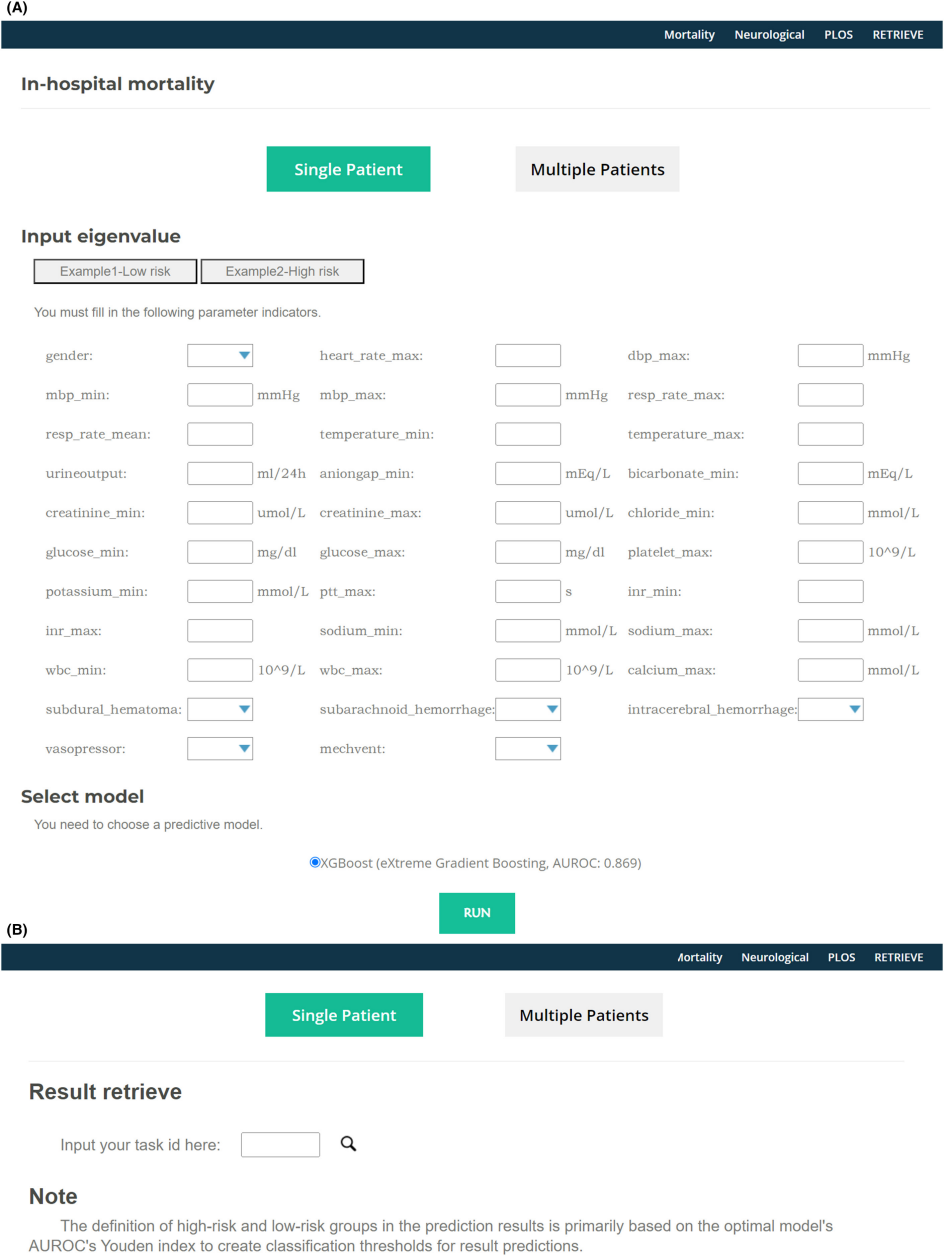
Online tools for diagnosing the three clinical outcomes of traumatic brain injury using the first 24 h of electronic health record clinical features. (A) Diagnosis page. (B) Results query page.

Considering the clinical utility and the fact that EHR data can provide performance superior to or even surpassing traditional scoring systems, we developed an emergency TBI prediction tool (www.tbi‐prophet.com) based on the optimal predictive model XGBoost and EHR features within 24 h for the three clinical outcomes. This system allows batch prediction and enables result queries based on automatically generated unique IDs. The system interface is illustrated in Figure 4.

## 4 DISCUSSION

Our research findings demonstrate the value of ML algorithms and routine collection of clinical data within 24 h during intensive care for predicting future clinical events in TBI patients. We observed that the models we constructed exhibited high predictive performance for the three clinical outcomes of interest: survival, neurological status, and PLOS. Such performance is not commonly observed in current clinical prognostic models, and we believe that the inclusion of PTS data and decoding of high‐frequency physiological data were key to this improvement. Our proposed approach has several advantages: We utilized a highly specific cohort of patients (i.e., critically ill patients with traumatic TBI) rather than a general population cohort; the data we included were based on routine clinical collection, eliminating the need for additional variables (e.g., the well‐known APACHE model for predicting ICU mortality, which requires collecting 142 variables); and the application of interpretable algorithms can provide insights for monitoring clinical states that change rapidly and aid in treatment decision‐making. Importantly, our analysis was conducted using data obtained through routine clinical practice, and we have developed simplified online tools with high predictive performance that can be utilized in clinical settings. This highlights the potential for validation on a larger scale and clinical implementation.

Some exploratory studies on feature analysis have indicated that a more detailed examination of physiological signals, such as curve shape, local averages, symmetry, mutations, and peak counts, can provide valuable information about the clinical trajectory of patients.^12^, ^13^, ^14^ Our research findings further underscore the potential hidden information of collecting PTS data within 24 hours of ICU admission. Although ML has been extensively developed in the prognosis of TBI patients, PTS has not yet been applied to this type of model.^15^, ^16^

The development and application of machine learning algorithms offer new opportunities for disease prediction in TBI patients, enabling informed decision‐making when sufficient and relevant data are available. We selected KNN, MLP, and XGBoost for our study due to their unique strengths and complementary characteristics. KNN is one of the simplest machine learning algorithms, which helps in understanding the inherent difficulty of the classification task.^17^ MLP, a type of neural network, excels at capturing complex nonlinear interactions in the data, making it well‐suited for predicting clinical outcomes where the relationships between variables are not straightforward.^18^ XGBoost, an ensemble learning method, is known for its robustness against overfitting, providing high predictive accuracy in various clinical applications.^19^, ^20^ This combination of models allows us to leverage their individual strengths for a comprehensive evaluation of our predictive tasks.

With the rapid development of artificial intelligence technology, the prediction of TBI‐related outcomes has entered a new era. Robson et al.^21^ utilized machine learning methods to predict early TBI mortality, achieving an AUC of 0.906. However, their model included multiple variables such as GCS scores, pupillary reactivity at admission, and CT scan results, which may not be feasible given the increasingly strained medical resources. Wu et al.^22^ included 2804 TBI patients to predict in‐hospital mortality and found that the XGBoost model could capture information hidden within the demographic and clinical predictive factors of TBI patients, resulting in more accurate predictions compared to the LR method. This is consistent with our findings, but their top predictive model requires inclusion of 54 clinical variables, significantly more than our study. Shikha et al.^23^ explored the value of predicting discharge neurological status based on imaging and clinical data, showing that the integration of multiple data sources achieved the best AUC value of 0.88. This is consistent with a major focus of our study, namely that the comprehensive exploration of diverse data can provide effective prognostic information. In our additional focus on PLOS outcomes, many studies similarly emphasize clinical characteristics associated with PLOS. For example, John et al.^24^ identified that the severity of TBI, age, and access to acute post‐care are independently associated with prolonged PLOS of ≥28 days, suggesting that high‐risk patients should be identified early to provide transitional care resources. This aligns with the aim of our study to provide a simplified automated diagnostic tool incorporating non‐human‐dependent predictive factors, maximizing predictive efficiency given the current constraints on medical resources and manpower.

In this study, all patients in the eICU‐CRD and MIMIC‐III databases were screened, and over 1000 TBI patients were adopted as the study subjects. Clinical outcome analysis and validation were conducted through ML technology and HTCSA analysis. One thing we would like to point out is that despite clinical guidelines, there is considerable variation in acute care for TBI patients among different institutions (even within the same ICU).^25^ We found a decrease in model recognition rates of 6%–10% in external validation in MIMIC III, which is not unexpected.

Given the highly variable and heterogeneous nature of individual clinical characteristics, the clinical interpretation of the features encompassed in risk assessment predictions will be crucial for achieving clinical applicability, necessitating transparency and traceability of the ML decision‐making process to be provided to physicians. In our study, we applied the SHAP algorithm to identify several well‐known predictive variables, as well as previously unreported variables. An interesting and important finding is that some EHR variables contribute significantly to our predictions for the three clinical outcomes. The clinical consensus for treating TBI suggests that the use of vasopressor agents can maintain appropriate cerebral perfusion pressure, reduce further injury, and improve patient prognosis and outcomes.^26^, ^27^ However, the use of mechanical ventilation may lead to ventilator‐associated pneumonia and potentially impact the patient's clinical progression.^28^, ^29^ Additionally, temperature control is necessary as abnormal body temperature can also affect the patient's neurological status.^30^, ^31^ Monitoring urine output is crucial as functional impairment and urodynamic abnormalities in TBI patients are associated with adverse outcomes.^32^ Moreover, elevated blood glucose levels in TBI patients may be related to severity and prognosis, necessitating the monitoring of cerebral glucose levels to predict the possibility of secondary ischemia and reflect treatment efficacy.^33^, ^34^ Furthermore, coagulation dysfunction is a common complication in TBI patients, and maintaining INR within normal levels contributes to improved treatment outcomes and prognosis.^35^, ^36^ These findings, to some extent, align with the current mainstream reports and indirectly validate the rationale behind the methodology we employed.

It should be noted that, in addition to the variables mentioned above, there may be other influential factors that hold particular significance for individual outcomes. For in‐hospital mortality, we identified subdural hematoma and white blood cell count as potential key factors. Similar to our findings, studies have also reported a strong association between subdural hematoma and increased mortality rate.^37^ Furthermore, elevated white blood cell count has been suggested as an indicator of severe inflammatory response, and thus, monitoring and better management of systemic infections may potentially reduce the mortality rate in TBI patients.^38^ Regarding the neurological functional status of TBI patients, we observed the critical roles of blood urea nitrogen (BUN) and SpO2. Acute kidney injury is one of the most common and severe complications following TBI.^39^ Previous research has indicated that intracranial pressure monitoring can guide the use of mannitol to protect renal function.^40^ Additionally, studies have found that the influence of TBI on mortality rate significantly changes with high oxygen levels.^41^ However, currently, there is no research exploring the relationship between BUN, SpO2, and the neurological functional status prognosis in TBI patients. PLOS is an important reflection of the severity of TBI. Some of the indicators we identified have received more attention in terms of their impact on TBI mortality, such as chloride,^42^ bicarbonate,^43^ and hemoglobin.^44^ The significance of these indicators for PLOS warrants further exploration.

Comprehensive monitoring and management of these key clinical features are crucial for the treatment of TBI patients. However, we want to emphasize that the relationships between the features indicated by our analysis and clinical outcomes are primarily correlational, and the implementation of appropriate intervention measures and confirmation of causal relationships require prospective studies to validate.

### 4.1 Limitations

Several limitations in this work need to be noted. Firstly, it is based on a retrospective analysis of past data, which may introduce bias and confounding factors, and the presence of unrecorded or missing data may impact the results. Secondly, the study only considered electronic medical record data within the first 24 h of admission and did not account for the influence of subsequent care intensity and life‐sustaining treatments during the treatment process. Furthermore, the effectiveness of machine learning algorithms is influenced by the size, quality, and complexity of the dataset and training models, and the decision‐making process can be difficult to interpret. Lastly, the predictive capability of machine learning algorithms may be limited when applied to new datasets, primarily due to significant differences in the treatment capabilities of hospitals at different levels, as well as the potential for the definition of PLOS to “float” with variations in the target population, necessitating additional cohort validation. Despite these limitations, this study employed SHAP for explanatory analysis and conducted external validation, providing new insights into the analysis and application of electronic medical records for TBI patients and holding potential to guide future clinical practice and decision‐making.

## 5 CONCLUSIONS

In conclusion, our findings demonstrate that ML models trained on EHR and PTS data can successfully differentiate inpatient survival, discharge neurological prognosis, and prolonged length of stay in TBI patients. The high‐resolution ICU time‐series data also contain valuable hidden information. Furthermore, our models are interpretable and reveal several potentially relevant prognostic features, warranting further exploration.

## AUTHOR CONTRIBUTIONS

Drs. Ding, Ms. Deng, and Mr. Wei contributed equally and share the first authorship.

RD and MD conceived and designed the study. HW applied for database and collected data. RD, MD, and HW drafted the manuscript, cleaned data, generated model, and did statistical analyses. YZ, GJ, LW, and HF revised the manuscript for important intellectual content. YZ, LW, HZ, and XH contributed to data interpretation. SZ and HY are the guarantors of the article, taking responsibility for the integrity of the work as a whole. All authors provided final approval of the version to be published.

## CONFLICT OF INTEREST STATEMENT

The authors declare that the research was conducted in the absence of any commercial or financial relationships that could be construed as a potential conflict of interest.

## PUBLISHER'S NOTE

All claims expressed in this article are solely those of the authors and do not necessarily represent those of their affiliated organizations or those of the publisher, the editors, and the reviewers. Any product that may be evaluated in this article, or claim that may be made by its manufacturer, is not guaranteed or endorsed by the publisher.

## Supporting information


Appendix S1.

## Data Availability

Publicly available datasets were analyzed in this study. These data can be found here: https://eicu‐crd.mit.edu/, https://mimic.mit.edu/.
