# Supplementary material for: Machine learning‐based prediction of clinical outcomes after traumatic brain injury: Hidden information of early physiological time series
Source: CNS Neurosci Ther. 2024 Jul 7;30(7):e14848. doi: 10.1111/cns.14848 (PMC11228354; doi:10.1111/cns.14848)
Supplement: Supplementary file 1 — Appendix S1. [file CNS-30-e14848-s001.docx]

Supplemental Materials

**Supplemental Methods**

**Data processing**

To mitigate the potential bias introduced by missing data, electronic health record (EHR) variables with a missing value proportion exceeding 30% were excluded from the final cohort. For the remaining variables, missing values were imputed using the missForest algorithm[1]. Furthermore, to address class imbalance, we employed the Synthetic Minority Oversampling Technique (SMOTE) algorithm, which increases the number of samples in the minority class through oversampling[2]. The final set of input features included 63 clinical characteristics of patients (Supplementary Table 1&2).

Subsequently, highly comparative time-series analysis (HCTSA), a MATLAB software package that has demonstrated successful applications across various fields[3], was utilized for processing the physiological time series (PTS) data. Based on HCTSA, over 7700 features were computed, encompassing distribution analysis, stationarity and step detection, fractal scaling, and other feature sets. Supplementary Table 3 provides a comprehensive summary of the HCTSA operations performed in this study.

Moreover, the eICU patient data was randomly divided into two distinct datasets: a training dataset comprising 70% of the patients, used for feature selection and model training, and a testing dataset used for evaluating the performance of the models, consisting of the remaining 30% of patients. The baseline characteristics for each modeling task across the training and test sets are presented in Supplementary Table 4.

**Feature selection**

To mitigate model complexity and uncover the underlying data patterns within each outcome, as well as to extract a feature subset containing maximum information content, lasso regularization was employed. The regularization coefficient lambda was determined through cross-validation, selecting the lambda value that yielded the best predictive performance. Moreover, when combining EHR and PTS, we assessed the multicollinearity among the selected features using the variance inflation factor (VIF), ensuring that the VIF values of the features included in the model training were all less than 10.

**Model Development**

Our study included three machine learning (ML) models: K-nearest neighbors (KNN), multi-layer perceptron (MLP), and extreme gradient boosting (XGBoost). For each of the three outcomes of interest, we trained these models using EHR data, PTS data, and a combination of both in the training set. To optimize the performance of the trained models, we conducted model tuning by adjusting predefined hyperparameters (Supplementary Table 5). Hyperparameter tuning was performed using 10-fold cross-validation within the training set loop. Furthermore, to assess the robustness of the models and methods employed, we conducted a 10-fold cross-validation with 50 repetitions on the entire dataset to validate and evaluate the external validation set.

**Model performance metrics and assessment**

For the trained models, the area under the receiver operating characteristic (ROC) curve, known as the AUROC, is chosen as the primary evaluation metric. The probability decision thresholds of the final models were determined based on Youden's index, which involves generating ROC curves for each final model, calculating Youden's index for various probability thresholds by adding sensitivity and specificity and subtracting 1, identifying the threshold corresponding to the maximum Youden's index as the optimal decision threshold, and utilizing this threshold to classify instances into positive or negative classes based on their predicted probabilities. In addition, our study encompasses a range of other common evaluation metrics, including decision curve analysis (DCA), sensitivity, specificity, F1 score, accuracy, positive predictive value (PPV) and negative predictive value (NPV) for each model. To ensure rigorous validation, assess the generalizability and robustness of the models, we conducted external validation using the MIMIC database. Additionally, we compared the optimal ML model with traditional scoring systems, including APACHE IV, APS III, GCS, and SOFA score.

**Model interpretation**

To ensure precise predictions, ML models leverage intricate algorithms and mathematical models. Nonetheless, interpreting the relevant features utilized in model construction can facilitate practical clinical implementation. In our study, we employed the Shapley Additive Explanations (SHAP) algorithm to compute the Shapley value for each feature, assigning an importance score to facilitate model interpretation and inform clinical decision-making.

**Statistical analysis**

All statistical analyses were performed in R (version 4.2.2), and two-sided P value, 0.05 was considered as statistically significant.

**References**

1. Stekhoven, D.J. and P. Buhlmann, *MissForest--non-parametric missing value imputation for mixed-type data.* Bioinformatics, 2012. **28**(1): p. 112-8.

2. Blagus, R. and L. Lusa, *SMOTE for high-dimensional class-imbalanced data.* BMC Bioinformatics, 2013. **14**: p. 106.

3. Fulcher, B.D. and N.S. Jones, *hctsa: A Computational Framework for Automated Time-Series Phenotyping Using Massive Feature Extraction.* Cell Syst, 2017. **5**(5): p. 527-531 e3.

**Supplemental Tables**

**Supplemental Table 1.** Electronic health record features across outcomes in eICU.

|  | In-hospital mortality | | Neurological outcome | | prolonged length of ICU stay | |
| --- | --- | --- | --- | --- | --- | --- |
|  | Expired (n=185) | Alive (n=825) | Unfavorable (n=278) | Favorable (n=739) | YES (n=218) | NO (n=607) |
| **Demographic** |  |  |  |  |  |  |
| Male | 107 (57.84 %) | 346 (41.94 %)* | 145 (52.16 %) | 308 (41.68 %) | 91 (41.74 %) | 255 (42.01 %) |
| **Intracranial injury types** |  |  |  |  |  |  |
| Epidural hematoma | 7 (3.78 %) | 34 (4.12 %) | 11 (3.96 %) | 31 (4.19 %) | 7 (3.21 %) | 27 (4.45 %) |
| Subdural hematoma | 93 (50.27 %) | 337 (40.85 %) | 123 (44.24 %) | 311 (42.08 %) | 85 (38.99 %) | 252 (41.52 %) |
| Subarachnoid hemorrhage | 77 (41.62 %) | 270 (32.73 %) | 109 (39.21 %) | 238 (32.21 %) | 79 (36.24 %) | 191 (31.47 %) |
| Intracerebral hemorrhage | 52 (28.11 %) | 135 (16.36 %)* | 69 (24.82 %) | 118 (15.97 %) | 39 (17.89 %) | 96 (15.82 %) |
| Cerebral contusion | 17 (9.19 %) | 40 (4.85 %) | 20 (7.19 %) | 37 (5.01 %) | 13 (5.96 %) | 27 (4.45 %) |
| **First day treatmen**t |  |  |  |  |  |  |
| Vasopressor | 43 (23.24 %) | 25 (3.03 %)* | 49 (17.63 %) | 19 (2.57 %)* | 16 (7.34 %) | 9 (1.48 %)* |
| Renal replacement therapy | 4 (2.16 %) | 5 (0.61 %) | 4 (1.44 %) | 5 (0.68 %) | 1 (0.46 %) | 4 (0.66 %) |
| Mechanical ventilation | 160 (86.49 %) | 275 (33.33 %)* | 218 (78.42 %) | 220 (29.77 %)* | 139 (63.76 %) | 136 (22.41 %)* |
| **Vital signs** |  |  |  |  |  |  |
| heart_rate_min | 67.65 (16.02) | 65.57 (13.35) | 67.54 (15.06) | 65.36 (13.35) | 68.98 (14.71) | 64.35 (12.62)* |
| heart_rate_max | 119.86 (28.54) | 103.42 (21.27)* | 117.58 (26.42) | 102.20 (20.98)* | 112.45 (22.17) | 100.19 (19.99)* |
| heart_rate_mean | 91.32 (18.14) | 81.30 (14.90)* | 89.92 (17.43) | 80.57 (14.63)* | 87.60 (16.39) | 79.05 (13.65)* |
| sbp_min | 92.08 (24.13) | 102.30 (17.25)* | 95.02 (22.92) | 102.42 (16.99)* | 99.10 (19.30) | 103.43 (16.33) |
| sbp_max | 164.34 (30.96) | 160.22 (23.76) | 165.47 (29.50) | 159.40 (23.20) | 163.01 (24.57) | 159.23 (23.41) |
| sbp_mean | 125.80 (17.69) | 129.11 (15.48) | 127.68 (17.35) | 128.88 (15.33) | 128.44 (16.12) | 129.35 (15.26) |
| dbp_min | 47.33 (12.87) | 51.74 (11.36)* | 48.42 (12.29) | 51.78 (11.50)* | 51.62 (11.76) | 51.79 (11.22) |
| dbp_max | 95.44 (23.83) | 97.16 (19.33) | 96.63 (23.11) | 96.99 (18.95) | 97.59 (19.11) | 97.01 (19.41) |
| dbp_mean | 66.69 (10.51) | 69.59 (10.03)* | 67.62 (10.24) | 69.54 (10.11) | 69.55 (10.08) | 69.60 (10.02) |
| mbp_min | 58.66 (17.67) | 66.47 (12.43)* | 60.31 (16.48) | 66.70 (12.33)* | 64.00 (13.24) | 67.36 (12.01) |
| mbp_max | 117.34 (24.92) | 111.45 (18.57) | 116.45 (23.74) | 111.09 (18.22)* | 111.53 (18.85) | 111.42 (18.48 |
| mbp_mean | 83.66 (10.88) | 85.67 (10.70) | 84.25 (10.89) | 85.67 (10.71) | 84.45 (10.20 | 86.12 (10.85) |
| resp_rate_min | 11.56 (5.32) | 11.98 (3.80) | 11.93 (4.73) | 11.88 (3.85) | 12.18 (3.98) | 11.90 (3.73) |
| resp_rate_max | 28.08 (6.91) | 28.00 (7.92) | 28.01 (7.26) | 28.01 (7.90) | 27.89 (7.09) | 28.04 (8.20) |
| resp_rate_mean | 19.19 (4.07) | 18.25 (3.34) | 18.90 (3.83) | 18.24 (3.36) | 18.38 (3.27) | 18.21 (3.37) |
| temperature_min | 35.59 (1.31) | 36.33 (0.75)* | 35.73 (1.39) | 36.36 (0.59)* | 36.23 (0.92) | 36.37 (0.68) |
| temperature_max | 38.14 (0.96) | 37.58 (0.67)* | 38.12 (0.92) | 37.52 (0.62)* | 38.02 (0.75) | 37.42 (0.57)* |
| temperature_mean | 37.00 (0.85) | 36.98 (0.49) | 37.06 (0.80) | 36.96 (0.45) | 37.19 (0.61) | 36.90 (0.41)* |
| spo2_min | 89.51 (15.71) | 90.96 (10.51) | 89.92 (14.69) | 90.99 (10.22) | 90.70 (11.35) | 91.05 (10.21) |
| spo2_max | 99.85 (0.61) | 99.53 (0.92)* | 99.84 (0.57) | 99.50 (0.95)* | 99.78 (0.62) | 99.45 (0.99)* |
| spo2_mean | 97.89 (2.19) | 97.29 (1.82) | 97.91 (2.09) | 97.22 (1.80)* | 97.94 (1.86) | 97.07 (1.76)* |
| Urineoutput | 3288.71 (2401.65) | 1958.51 (1539.69)* | 2914.61 (2281.93) | 1936.74 (1510.35)* | 2358.16 (1890.43) | 1810.97 (1360.60)* |
| **Laboratory results** |  |  |  |  |  |  |
| aniongap_min | 9.68 (4.52) | 10.51 (4.21) | 9.79 (4.30) | 10.57 (4.24) | 10.78 (4.31) | 10.41 (4.17) |
| aniongap_max | 13.84 (5.16) | 12.58 (4.77) | 13.39 (4.92) | 12.62 (4.82) | 13.19 (4.58) | 12.36 (4.83) |
| bicarbonate_min | 20.13 (4.64) | 23.18 (3.66)* | 20.92 (4.36) | 23.27 (3.68)* | 21.96 (3.66) | 23.64 (3.56)* |
| bicarbonate_max | 24.08 (3.64) | 25.24 (3.29)* | 24.39 (3.44) | 25.27 (3.32)* | 24.33 (3.53) | 25.59 (3.13)* |
| creatinine_min | 1.21 (1.47) | 0.90 (0.74) | 1.14 (1.29) | 0.92 (0.86) | 0.90 (0.64) | 0.90 (0.77) |
| creatinine_max | 1.50 (1.57) | 1.03 (0.83)* | 1.38 (1.43) | 1.05 (0.96)* | 1.03 (0.65) | 1.03 (0.88) |
| chloride_min | 102.96 (7.30) | 102.98 (5.62) | 102.92 (6.86) | 102.98 (5.58) | 103.89 (6.52) | 102.64 (5.22) |
| chloride_max | 110.40 (9.20) | 105.98 (5.63)* | 109.46 (8.67) | 105.79 (5.36)* | 107.94 (6.82) | 105.26 (4.93)* |
| glucose_min | 133.61 (46.67) | 117.87 (33.77)* | 130.84 (42.15) | 117.08 (34.04)* | 124.90 (31.48) | 115.26 (34.24)* |
| glucose_max | 199.91 (74.26) | 145.23 (60.34)* | 188.03 (70.91) | 143.24 (60.59)* | 160.30 (57.74) | 139.65 (60.38)* |
| hematocrit_min | 30.95 (8.09) | 34.55 (6.61)* | 31.38 (7.62) | 34.80 (6.58)* | 32.02 (7.32) | 35.48 (6.07)* |
| hematocrit_max | 37.57 (6.16) | 38.47 (5.77) | 37.60 (5.72) | 38.55 (5.87) | 37.85 (6.00) | 38.70 (5.67) |
| hemoglobin_min | 10.39 (2.79) | 11.64 (2.26* | 10.53 (2.61) | 11.73 (2.25)* | 10.83 (2.45) | 11.93 (2.11)* |
| hemoglobin_max | 12.61 (2.20) | 12.96 (2.04) | 12.62 (2.05) | 13.00 (2.07) | 12.82 (2.08) | 13.02 (2.03) |
| platelet_min | 169.01 (123.26) | 192.65 (76.22) | 179.77 (113.02) | 191.52 (75.20) | 183.93 (80.36) | 195.89 (74.44) |
| platelet_max | 216.40 (121.75) | 223.36 (83.18) | 226.36 (116.65) | 220.53 (80.12) | 231.63 (93.50) | 220.29 (78.87) |
| potassium_min | 3.49 (0.50) | 3.74 (0.48)* | 3.52 (0.51) | 3.76 (0.48)* | 3.64 (0.52) | 3.77 (0.47) |
| potassium_max | 4.44 (0.69) | 4.18 (0.54)* | 4.37 (0.65) | 4.18 (0.54)* | 4.22 (0.55) | 4.16 (0.53) |
| ptt_min | 29.52 (6.73) | 28.07 (6.52) | 29.16 (6.67) | 28.03 (6.50) | 27.16 (5.09) | 28.45 (7.00) |
| ptt_max | 35.88 (17.03) | 29.00 (7.30)* | 33.85 (14.86) | 28.94 (7.38)* | 28.55 (5.76) | 29.19 (7.84) |
| inr_min | 1.21 (0.34) | 1.10 (0.18)* | 1.19 (0.33) | 1.09 (0.15) | 1.12 (0.20) | 1.09 (0.17) |
| inr_max | 1.69 (1.35) | 1.23 (0.55)* | 1.60 (1.20) | 1.21 (0.53)* | 1.33 (0.82) | 1.19 (0.41) |
| pt_min | 14.33 (3.65) | 13.29 (2.15)* | 14.22 (3.54) | 13.21 (1.92)* | 13.48 (2.38) | 13.22 (2.06) |
| pt_max | 19.11 (13.28) | 14.46 (5.03)* | 18.17 (11.71) | 14.28 (4.78)* | 15.42 (7.04) | 14.11 (4.00) |
| sodium_min | 137.18 (5.75) | 137.91 (4.40) | 137.22 (5.41) | 137.96 (4.36) | 138.33 (4.74) | 137.75 (4.26) |
| sodium_max | 143.84 (7.36) | 140.51 (4.25)* | 143.10 (6.78) | 140.37 (4.09)* | 141.81 (4.73) | 140.02 (3.95)* |
| bun_min | 17.51 (11.83) | 15.31 (10.61) | 17.20 (11.60) | 15.26 (10.72) | 15.17 (11.50) | 15.37 (10.27) |
| bun_max | 21.44 (13.59) | 18.05 (11.62) | 20.98 (13.41) | 17.95 (11.69) | 18.07 (11.75) | 18.04 (11.58) |
| wbc_min | 11.21 (5.51) | 9.73 (3.93)* | 11.01 (5.11) | 9.63 (3.90)* | 10.45 (4.10) | 9.46 (3.84) |
| wbc_max | 16.42 (8.30) | 12.50 (5.61)* | 15.56 (7.49) | 12.37 (5.67)* | 14.43 (6.15) | 11.78 (5.22)* |
| calcium_min | 7.68 (1.18) | 8.33 (0.86)* | 7.80 (1.13) | 8.36 (0.84)* | 7.97 (0.89) | 8.46 (0.82)* |
| calcium_max | 8.63 (0.78) | 8.79 (0.69) | 8.64 (0.77) | 8.81 (0.69) | 8.58 (0.76) | 8.87 (0.65)* |

Data are n (%) or mean (SD). Two-tailed Student’s t-tests or Mann-Whitney U-test for continuous variables, Chi-squared or Fisher’s exact test for categorical variables. *=*P*<0.05. SBP: systolic blood pressure; DBP: diastolic blood pressure; MBP: mean blood pressure; SpO2: oxygen saturation; PTT: partial thromboplastin time; INR: International Normalized Ratio; PT: prothrombin time; BUN: blood urea nitrogen; WBC: white blood cell.

**Supplemental Table 2.** Electronic health record features across outcomes in Medical Information Mart for Intensive Care III.

|  | In-hospital mortality | | Neurological outcome | | prolonged length of ICU stay | |
| --- | --- | --- | --- | --- | --- | --- |
|  | Expired (n=29) | Alive (n=39) | Unfavorable (n=35) | Favorable (n=33) | YES (n=10) | NO (n=29) |
| **Demographic** |  |  |  |  |  |  |
| Male | 17 (58.62 %) | 32 (82.05 %) | 22 (62.86 %) | 27 (81.82 %) | 8 (80.00 %) | 24 (82.76 %) |
| **Intracranial injury types** |  |  |  |  |  |  |
| Epidural hematoma | 7 (24.14 %) | 9 (23.08 %) | 8 (22.86 %) | 8 (24.24 %) | 2 (20.00 %) | 7 (24.14 %) |
| Subdural hematoma | 7 (24.14 %) | 9 (23.08 %) | 8 (22.86 %) | 8 (24.24 %) | 2 (20.00 %) | 7 (24.14 %) |
| Subarachnoid hemorrhage | 7 (24.14 %) | 9 (23.08 %) | 8 (22.86 %) | 8 (24.24 %) | 2 (20.00 %) | 7 (24.14 %) |
| Intracerebral hemorrhage | 0 (0 %) | 0 (0 %) | 0 (0 %) | 0 (0 %) | 0 (0 %) | 0 (0 %) |
| Cerebral contusion | 6 (20.69 %) | 7 (17.95 %) | 8 (22.86 %) | 5 (15.15 %) | 3 (30.00 %) | 4 (13.79 %) |
| **First day treatmen**t |  |  |  |  |  |  |
| Vasopressor | 17 (58.62 %) | 9 (23.08 %) | 19 (54.29 %) | 7 (21.21 %) | 8 (80.00 %) | 1 (3.45 %)* |
| Renal replacement therapy | 0 (0.00 %) | 1 (2.56 %) | 0 (0.00 %) | 1 (3.03 %) | 0 (0.00 %) | 1 (3.45 %) |
| Mechanical ventilation | 26 (89.66 %) | 21 (53.85 %) | 30 (85.71 %) | 17 (51.52 %) | 8 (80.00 %) | 13 (44.83 %) |
| **Vital signs** |  |  |  |  |  |  |
| heart_rate_min | 68.55 (18.26) | 66.62 (14.68) | 69.74 (17.39) | 65.00 (14.72) | 63.10 (14.59) | 67.83 (14.77) |
| heart_rate_max | 110.38 (27.59) | 107.54 (21.76) | 110.77 (25.43) | 106.61 (23.16) | 119.30 (27.01) | 103.48 (18.49) |
| heart_rate_mean | 87.98 (17.59) | 83.63 (14.69) | 88.51 (16.50) | 82.28 (15.06) | 87.21 (11.55) | 82.40 (15.61) |
| sbp_min | 88.76 (14.50) | 97.44 (14.07) | 92.09 (15.67) | 95.48 (13.83) | 88.40 (15.04) | 100.55 (12.53) |
| sbp_max | 160.10 (20.21) | 155.38 (23.20) | 161.26 (21.14) | 153.30 (22.35) | 165.20 (24.42) | 152.00 (22.19) |
| sbp_mean | 123.00 (13.47) | 124.42 (16.69) | 125.48 (15.78) | 122.04 (14.83) | 122.96 (19.92) | 124.92 (15.80) |
| dbp_min | 43.31 (11.67) | 46.08 (10.67) | 44.51 (11.14) | 45.30 (11.24) | 41.40 (9.28) | 47.69 (10.79) |
| dbp_max | 89.69 (19.77) | 88.44 (17.64) | 89.20 (18.87) | 88.73 (18.27) | 95.30 (25.57) | 86.07 (13.77) |
| dbp_mean | 62.60 (9.54) | 62.99 (10.37) | 63.09 (9.61) | 62.54 (10.44) | 60.42 (9.81) | 63.87 (10.57) |
| mbp_min | 54.89 (14.33) | 58.79 (13.53) | 56.85 (13.76) | 57.42 (14.26) | 56.90 (9.21) | 59.45 (14.81) |
| mbp_max | 109.20 (18.73) | 110.87 (31.46) | 109.45 (18.33) | 110.91 (33.55) | 123.10 (21.93) | 106.66 (33.42) |
| mbp_mean | 80.23 (9.46) | 80.00 (10.26) | 81.32 (9.95) | 78.80 (9.74) | 80.05 (10.79) | 79.99 (10.27) |
| resp_rate_min | 13.03 (4.23) | 11.56 (3.50) | 13.00 (4.30) | 11.33 (3.20) | 10.80 (3.71) | 11.83 (3.45) |
| resp_rate_max | 28.28 (5.95) | 25.41 (4.64) | 28.11 (5.60) | 25.06 (4.74) | 26.30 (5.81) | 25.10 (4.24) |
| resp_rate_mean | 19.75 (3.86) | 18.47 (3.43) | 19.83 (3.67) | 18.15 (3.47) | 19.08 (3.63) | 18.25 (3.40) |
| temperature_min | 35.49 (2.29) | 36.35 (0.66 | 35.67 (2.14) | 36.32 (0.62) | 36.26 (0.97) | 36.38 (0.53) |
| temperature_max | 38.12 (1.16) | 37.84 (0.65) | 38.14 (1.09) | 37.76 (0.62) | 38.17 (0.45) | 37.73 (0.67) |
| temperature_mean | 37.00 (1.13) | 37.15 (0.50) | 37.08 (1.08) | 37.09 (0.44) | 37.26 (0.58) | 37.11 (0.47) |
| spo2_min | 92.03 (9.28) | 92.95 (6.51) | 92.54 (8.64) | 92.58 (6.84) | 90.70 (11.39) | 93.72 (3.65) |
| spo2_max | 100.00 (0.00) | 99.82 (0.39) | 100.00 (0.00) | 99.79 (0.42) | 99.80 (0.42) | 99.83 (0.38) |
| spo2_mean | 98.36 (2.30) | 97.62 (1.57) | 98.42 (2.15) | 97.42 (1.55) | 97.86 (1.39) | 97.54 (1.65) |
| Urineoutput | 2982.57 (1842.24) | 3204.74 (7040.15) | 2858.47 (1781.26) | 3378.25 (7656.18) | 1324.30 (285.42) | 3876.32 (8131.19) |
| **Laboratory results** |  |  |  |  |  |  |
| aniongap_min | 13.18 (2.14) | 12.76 (2.15) | 12.97 (2.05) | 12.91 (2.26) | 13.40 (2.22) | 12.54 (2.12) |
| aniongap_max | 16.93 (3.24) | 15.42 (3.19) | 16.61 (3.15) | 15.52 (3.36) | 16.00 (2.71) | 15.21 (3.37) |
| bicarbonate_min | 21.39 (4.05) | 23.95 (4.55) | 21.70 (4.14) | 24.03 (4.59) | 22.10 (3.90) | 24.61 (4.65) |
| bicarbonate_max | 24.25 (4.22) | 26.66 (4.19) | 24.45 (4.16) | 26.82 (4.25) | 24.10 (3.57) | 27.57 (4.06) |
| creatinine_min | 1.00 (0.39) | 1.19 (1.03) | 0.97 (0.37) | 1.25 (1.10) | 1.05 (0.37) | 1.24 (1.18) |
| creatinine_max | 1.37 (0.64) | 1.38 (1.16) | 1.30 (0.61) | 1.45 (1.24) | 1.27 (0.49) | 1.41 (1.31) |
| chloride_min | 102.79 (6.01) | 102.10 (5.09) | 102.74 (6.13) | 102.03 (4.73) | 102.60 (5.17) | 101.93 (5.14) |
| chloride_max | 110.41 (6.51) | 105.95 (6.17) | 109.71 (7.09) | 105.88 (5.60) | 106.10 (5.59) | 105.90 (6.46) |
| glucose_min | 130.41 (42.25) | 125.56 (47.78) | 133.66 (41.20) | 121.24 (49.00) | 153.00 (50.22) | 116.10 (43.89) |
| glucose_max | 207.86 (69.67) | 163.38 (57.83) | 203.31 (64.68) | 160.12 (61.75) | 207.20 (48.19) | 148.28 (53.57) |
| hematocrit_min | 28.00 (7.30) | 32.36 (6.41) | 28.32 (6.87) | 32.82 (6.67) | 29.18 (5.77) | 33.46 (6.35) |
| hematocrit_max | 37.17 (6.55) | 37.05 (6.30) | 36.80 (6.08) | 37.42 (6.72) | 36.19 (5.13) | 37.34 (6.71) |
| hemoglobin_min | 9.76 (2.40) | 11.09 (2.35) | 9.81 (2.25) | 11.28 (2.45) | 9.85 (1.96) | 11.52 (2.35) |
| hemoglobin_max | 12.51 (2.13) | 12.47 (2.21) | 12.37 (1.98) | 12.62 (2.37) | 11.96 (1.62) | 12.65 (2.38) |
| platelet_min | 181.41 (94.26) | 197.05 (70.06) | 190.29 (94.40) | 190.48 (65.28) | 187.90 (66.61) | 200.21 (72.08) |
| platelet_max | 251.07 (162.82) | 245.18 (83.37) | 255.46 (152.56) | 239.45 (81.25) | 246.80 (82.40) | 244.62 (85.14) |
| potassium_min | 3.61 (0.69) | 3.73 (0.48) | 3.62 (0.66) | 3.75 (0.48) | 3.81 (0.51) | 3.70 (0.48) |
| potassium_max | 4.70 (0.72) | 4.21 (0.52) | 4.61 (0.71) | 4.22 (0.53) | 4.33 (0.78) | 4.17 (0.40) |
| ptt_min | 26.06 (5.03) | 26.83 (4.14) | 25.85 (4.70) | 27.19 (4.29) | 23.94 (3.00) | 27.83 (4.04) |
| ptt_max | 33.71 (16.31) | 30.60 (9.17) | 32.59 (15.04) | 31.22 (9.80) | 28.81 (5.15) | 31.22 (10.20) |
| inr_min | 1.19 (0.24) | 1.13 (0.16) | 1.19 (0.22) | 1.12 (0.17) | 1.14 (0.14) | 1.12 (0.17) |
| inr_max | 1.49 (0.74) | 1.32 (0.45) | 1.48 (0.69) | 1.30 (0.46) | 1.38 (0.34) | 1.30 (0.49) |
| pt_min | 12.88 (1.66) | 12.81 (1.66) | 12.93 (1.56) | 12.74 (1.75) | 12.96 (1.18) | 12.76 (1.81) |
| pt_max | 15.16 (4.39) | 14.46 (4.20) | 15.17 (4.24) | 14.33 (4.31) | 14.79 (3.09) | 14.35 (4.56) |
| sodium_min | 136.62 (5.65) | 137.67 (4.46) | 136.69 (5.33) | 137.79 (4.61) | 135.40 (2.91) | 138.45 (4.66) |
| sodium_max | 144.17 (5.31) | 141.51 (4.04) | 143.54 (5.43) | 141.70 (3.83) | 139.80 (3.16) | 142.10 (4.19) |
| bun_min | 21.21 (15.36) | 18.97 (14.44) | 20.03 (14.22) | 19.82 (15.55) | 23.00 (14.38) | 17.59 (14.45) |
| bun_max | 26.69 (17.91) | 22.15 (15.07) | 25.06 (16.71) | 23.06 (16.19) | 27.60 (15.21) | 20.28 (14.82) |
| wbc_min | 10.92 (4.90) | 9.96 (4.20) | 10.92 (4.56) | 9.78 (4.44) | 11.41 (4.31) | 9.46 (4.12) |
| wbc_max | 18.17 (10.80) | 13.82 (5.98) | 17.43 (10.01) | 13.82 (6.40) | 16.96 (6.71) | 12.74 (5.42) |
| calcium_min | 0.98 (0.20) | 1.06 (0.10) | 0.98 (0.19) | 1.07 (0.11) | 1.03 (0.12) | 1.09 (0.09) |
| calcium_max | 1.24 (0.17) | 1.12 (0.09) | 1.23 (0.16) | 1.11 (0.09) | 1.12 (0.09) | 1.12 (0.09) |

Data are n (%) or mean (SD). Two-tailed Student’s t-tests or Mann-Whitney U-test for continuous variables, Chi-squared or Fisher’s exact test for categorical variables. *=*P*<0.05. SBP: systolic blood pressure; DBP: diastolic blood pressure; MBP: mean blood pressure; SpO2: oxygen saturation; PTT: partial thromboplastin time; INR: International Normalized Ratio; PT: prothrombin time; BUN: blood urea nitrogen; WBC: white blood cell.

**Supplemental Table 3.** Summary for highly comparative time-series analysis operations.

| **Distribution** | | | |
| --- | --- | --- | --- |
| DN_Burstiness | DN_CompareKSFit | DN_CustomSkewness | DN_FitKernelSmooth |
| DN_Fit_mle | DN_HighLowMu | DN_HistogramMode | DN_Mean |
| DN_MinMax | DN_Moments | DN_OutlierInclude | DN_OutlierTest |
| DN_ProportionValues | DN_Quantile | DN_RemovePoints | DN_SimpleFit |
| DN_Spread | DN_TrimmedMean | DN_HistogramAsymmetry | DN_Unique |
| DN_Withinp | DN_cv | DN_pleft | EN_DistributionEntropy |
| HT_DistributionTest |  |  |  |
| **Correlation** | | | |
| CO_AddNoise | CO_AutoCorr | CO_AutoCorrShape | CO_Embed2 |
| CO_Embed2_AngleTau | CO_Embed2_Basic | CO_Embed2_Dist | CO_Embed2_Shapes |
| CO_FirstCrossing | CO_FirstMin | CO_NonlinearAutocorr | CO_StickAngles |
| CO_TranslateShape | CO_f1ecac | CO_fzcglscf | CO_glscf |
| CO_tc3 | CO_trev | DK_crinkle | DK_theilerQ |
| DK_timerev | NL_embed_PCA | CO_RM_AMInformation | CO_CompareMinAMI |
| CO_HistogramAMI | IN_AutoMutualInfoStats |  |  |
| **Information Theory** | | | |
| EN_ApEn | EN_CID | EN_MS_LZcomplexity | EN_MS_shannon |
| EN_PermEn | EN_RM_entropy | EN_Randomize | EN_SampEn |
| EN_mse | EN_rpde | EN_wentropy |  |
| **Time-series model fitting and forecasting** | | | |
| MF_ARMA_orders | MF_AR_arcov | MF_CompareAR | MF_CompareTestSets |
| MF_ExpSmoothing | MF_FitSubsegments | MF_GARCHcompare | MF_GARCHfit |
| MF_GP_FitAcross | MF_GP_LocalPrediction | MF_GP_hyperparameters | MF_StateSpaceCompOrder |
| MF_StateSpace_n4sid | MF_arfit | MF_armax | MF_hmm_CompareNStates |
| MF_hmm_fit | MF_steps_ahead | FC_LocalSimple | FC_LoopLocalSimple |
| FC_Surprise | PP_ModelFit |  |  |
| **Stationarity and step detection** | | | |
| SY_DriftingMean | SY_DynWin | SY_KPSStest | SY_LocalDistributions |
| SY_LocalGlobal | SY_PPtest | SY_RangeEvolve | SY_SlidingWindow |
| SY_SpreadRandomLocal | SY_StatAv | SY_StdNthDer | SY_StdNthDerChange |
| SY_TISEAN_nstat_z | SY_VarRatioTest | CP_ML_StepDetect | CP_l1pwc_sweep_lambda |
| CP_wavelet_varchg |  |  |  |
| **Nonlinear time-series analysis and fractal scaling** | | | |
| NL_BoxCorrDim | NL_DVV | NL_MS_fnn | NL_MS_nlpe |
| NL_TISEAN_c1 | NL_TISEAN_d2 | NL_TISEAN_fnn | NL_TSTL_FractalDimensions |
| NL_TSTL_GPCorrSum | NL_TSTL_LargestLyap | NL_TSTL_PoincareSection | NL_TSTL_ReturnTime |
| NL_TSTL_TakensEstimator | NL_TSTL_acp | NL_TSTL_dimensions | NL_crptool_fnn |
| SD_SurrogateTest | SD_TSTL_surrogates | TSTL_delaytime | TSTL_localdensity |
| NL_nsamdf | SC_MMA | SC_fastdfa | SC_FluctAnal |
| **Fourier and wavelet transforms, periodicity measures** | | | |
| SP_Summaries | DT_IsSeasonal | PD_PeriodicityWang | WL_DetailCoeffs |
| WL_coeffs | WL_cwt | WL_dwtcoeff | WL_fBM |
| WL_scal2frq |  |  |  |
| **Symbolic transformations** | | | |
| SB_BinaryStats | SB_BinaryStretch | SB_MotifThree | SB_MotifTwo |
| SB_TransitionMatrix | SB_TransitionpAlphabet |  |  |
| **Statistics from biomedical signal processing** | | | |
| MD_hrv_classic | MD_pNN | MD_polvar | MD_rawHRVmeas |
| **Basic statistics, trend** | | | |
| SY_Trend | ST_FitPolynomial | ST_Length | ST_LocalExtrema |
| ST_MomentCorr | ST_SimpleStats |  |  |
| **Others** | | | |
| EX_MovingThreshold | HT_HypothesisTest | NW_VisibilityGraph | PH_ForcePotential |
| PH_Walker | PP_Compare | PP_Iterate |  |

**Supplemental Table 4.** Electronic health record features across training and test sets for each modelling task.

|  | In-hospital mortality | | Neurological outcome | | prolonged length of ICU stay | |
| --- | --- | --- | --- | --- | --- | --- |
|  | Training set (n=707) | Testing set (n=303) | Training set (n=711) | Testing set (n=306) | Training set (n=577) | Testing set (n=248) |
| Male | 319 (45.12%) | 134 (44.22%) | 319 (44.87%) | 134 (43.79%) | 247 (42.81 %) | 99 (39.92 %) |
| Intracranial injury types |  |  |  |  |  |  |
| Epidural hematoma | 34 (4.81%) | 7 (2.31%) | 29 (4.08%) | 13 (4.25%) | 22 (3.81 %) | 12 (4.84 %) |
| Subdural hematoma | 306 (43.28%) | 124 (40.92%) | 304 (42.76%) | 130 (42.48%) | 240 (41.59 %) | 97 (39.11 %) |
| Subarachnoid hemorrhage | 255 (36.07%) | 92 (30.36%) | 244 (34.32%) | 103 (33.66%) | 191 (33.10 %) | 79 (31.85 %) |
| Intracerebral hemorrhage | 125 (17.68%) | 62 (20.46%) | 136 (19.13%) | 51 (16.67%) | 93 (16.12 %) | 42 (16.94 %) |
| Cerebral contusion | 37 (5.23%) | 20 (6.60%) | 42 (5.91%) | 15 (4.90%) | 30 (5.20 %) | 10 (4.03 %) |
| First day treatment |  |  |  |  |  |  |
| Vasopressor | 46 (6.51%) | 22 (7.26%) | 50 (7.03%) | 18 (5.88%) | 21 (3.64 %) | 4 (1.61 %) |
| Renal replacement therapy | 5 (0.71%) | 4 (1.32%) | 7 (0.98%) | 2 (0.65%) | 5 (0.87 %) | 0 (0.00 %) |
| Mechanical ventilation | 303 (42.86%) | 132 (43.56%) | 306 (43.04%) | 132 (43.14%) | 190 (32.93 %) | 85 (34.27 %) |
| Vital signs |  |  |  |  |  |  |
| heart_rate_min | 66.02 (14.37) | 65.79 (12.74) | 65.83 (14.10) | 66.25 (13.31) | 65.29 (12.92) | 66.23 (14.32) |
| heart_rate_max | 106.87 (24.19) | 105.36 (22.24) | 106.35 (23.72) | 106.50 (23.35) | 103.30 (21.76) | 103.72 (20.12) |
| heart_rate_mean | 83.27 (16.53) | 82.79 (14.71) | 83.10 (16.45) | 83.17 (14.87) | 81.05 (14.75) | 81.89 (15.23) |
| sbp_min | 100.66 (18.69) | 99.96 (19.97) | 100.47 (18.92) | 100.27 (19.41) | 102.92 (17.60) | 100.84 (16.34) |
| sbp_max | 162.12 (25.67) | 158.26 (24.05) | 160.93 (25.93) | 161.35 (23.43) | 160.70 (23.84) | 159.09 (23.57) |
| sbp_mean | 128.93 (15.75) | 127.53 (16.37) | 128.45 (16.06) | 128.80 (15.55) | 129.68 (15.84) | 127.79 (14.56) |
| dbp_min | 51.03 (11.48) | 50.76 (12.43) | 50.79 (11.75) | 51.05 (11.98) | 52.05 (11.54) | 51.04 (10.92) |
| dbp_max | 97.48 (20.24) | 95.37 (20.09) | 96.97 (20.78) | 96.71 (18.64) | 96.73 (19.22) | 98.16 (19.57) |
| dbp_mean | 69.36 (10.07) | 68.37 (10.39) | 69.00 (10.31) | 69.05 (9.88) | 69.76 (10.31) | 69.19 (9.36) |
| mbp_min | 65.04 (13.48) | 65.05 (14.73) | 64.91 (13.83) | 65.12 (13.99) | 66.92 (12.42) | 65.41 (12.40) |
| mbp_max | 113.10 (19.47) | 111.19 (21.15) | 112.47 (20.61) | 112.70 (18.51) | 111.70 (18.72) | 110.87 (18.22) |
| mbp_mean | 85.60 (10.54) | 84.62 (11.24) | 85.28 (10.99) | 85.28 (10.27) | 86.03 (10.90) | 84.86 (10.20) |
| resp_rate_min | 11.86 (4.23) | 11.99 (3.85) | 11.80 (4.24) | 12.12 (3.78) | 11.97 (3.89) | 11.98 (3.59) |
| resp_rate_max | 28.01 (7.85) | 28.02 (7.50) | 28.06 (7.97) | 27.90 (7.14) | 27.97 (8.01) | 28.08 (7.71) |
| resp_rate_mean | 18.38 (3.46) | 18.51 (3.59) | 18.35 (3.58) | 18.57 (3.33) | 18.21 (3.47) | 18.34 (3.02) |
| temperature_min | 36.18 (0.94) | 36.22 (0.89) | 36.18 (0.94) | 36.22 (0.89) | 36.33 (0.65) | 36.32 (0.95) |
| temperature_max | 37.68 (0.78) | 37.70 (0.74) | 37.67 (0.77) | 37.72 (0.76) | 37.57 (0.67) | 37.61 (0.69) |
| temperature_mean | 36.98 (0.57) | 37.00 (0.58) | 36.97 (0.55) | 37.01 (0.61) | 36.97 (0.47) | 36.99 (0.52) |
| spo2_min | 90.65 (12.09) | 90.84 (10.35) | 90.71 (11.63) | 90.71 (11.40) | 90.93 (11.19) | 91.04 (8.72) |
| spo2_max | 99.58 (0.89) | 99.60 (0.87) | 99.60 (0.88) | 99.57 (0.87) | 99.51 (0.93) | 99.59 (0.89) |
| spo2_mean | 97.40 (1.92) | 97.38 (1.87) | 97.43 (1.92) | 97.33 (1.86) | 97.29 (1.86 | 97.28 (1.74) |
| Urineoutput | 2220.49 (1862.73) | 2143.04 (1642.86) | 2228.23 (1858.21) | 2135.95 (1672.92) | 1937.29 (1593.37) | 2007.45 (1410.14) |
| Laboratory results |  |  |  |  |  |  |
| aniongap_min | 10.28 (4.29) | 10.54 (4.26) | 10.43 (4.30) | 10.19 (4.19) | 10.43 (4.24) | 10.68 (4.14) |
| aniongap_max | 12.80 (4.84) | 12.85 (4.95) | 12.88 (4.79) | 12.70 (5.01) | 12.41 (4.80) | 12.99 (4.71) |
| bicarbonate_min | 22.61 (3.98) | 22.65 (4.16) | 22.65 (4.09) | 22.55 (3.85) | 23.26 (3.68) | 22.98 (3.63) |
| bicarbonate_max | 24.98 (3.32) | 25.15 (3.54) | 25.06 (3.52) | 24.94 (3.01) | 25.31 (3.28) | 25.09 (3.33) |
| creatinine_min | 0.97 (1.02) | 0.94 (0.66) | 1.01 (1.11) | 0.91 (0.68) | 0.92 (0.81) | 0.85 (0.51) |
| creatinine_max | 1.12 (1.11) | 1.11 (0.80) | 1.17 (1.24) | 1.06 (0.78) | 1.06 (0.91) | 0.97 (0.58) |
| chloride_min | 102.87 (6.09) | 103.24 (5.67) | 102.70 (5.88) | 103.59 (6.11) | 103.14 (5.36) | 102.61 (6.21) |
| chloride_max | 106.83 (6.73) | 106.75 (6.53) | 106.62 (6.73) | 107.26 (6.44) | 106.13 (5.73) | 105.64 (5.36) |
| glucose_min | 120.40 (36.21) | 121.76 (38.88) | 120.48 (37.36) | 121.85 (36.07) | 117.62 (32.44) | 118.44 (36.76) |
| glucose_max | 154.36 (67.29) | 157.95 (65.05) | 154.62 (62.80) | 158.02 (74.90) | 144.66 (54.55) | 146.59 (72.22) |
| hematocrit_min | 33.65 (7.12) | 34.43 (6.84) | 33.56 (7.16 | 34.53 (6.73 | 34.30 (6.58) | 35.12 (6.65) |
| hematocrit_max | 38.15 (5.87) | 38.66 (5.80) | 38.10 (5.83 | 38.71 (5.86 | 38.25 (5.86) | 38.97 (5.54) |
| hemoglobin_min | 11.33 (2.44) | 11.58 (2.35) | 11.30 (2.45 | 11.62 (2.31 | 11.54 (2.24) | 11.85 (2.29) |
| hemoglobin_max | 12.84 (2.09) | 13.04 (2.05) | 12.83 (2.07 | 13.03 (2.07 | 12.89 (2.07) | 13.14 (1.98) |
| platelet_min | 186.94 (73.67) | 191.36 (113.54) | 187.00 (91.52 | 191.14 (77.34 | 192.23 (76.45) | 193.67 (75.83) |
| platelet_max | 221.91 (79.94) | 222.44 (114.77) | 222.16 (96.71 | 222.10 (79.10 | 222.86 (81.02) | 224.54 (88.25) |
| potassium_min | 3.69 (0.51) | 3.70 (0.47) | 3.68 (0.51 | 3.72 (0.47 | 3.75 (0.48) | 3.71 (0.49) |
| potassium_max | 4.23 (0.59) | 4.22 (0.56) | 4.21 (0.58 | 4.27 (0.58 | 4.19 (0.53) | 4.15 (0.55) |
| ptt_min | 28.44 (6.81) | 28.26 (6.00) | 28.59 (6.87 | 27.89 (5.73 | 28.15 (6.81) | 27.86 (5.69) |
| ptt_max | 30.40 (10.19) | 30.80 (11.70) | 30.82 (11.53 | 29.65 (7.62 | 29.09 (7.71) | 28.76 (6.04) |
| inr_min | 1.12 (0.25) | 1.13 (0.17) | 1.13 (0.24 | 1.11 (0.19 | 1.10 (0.17) | 1.10 (0.19) |
| inr_max | 1.32 (0.87) | 1.34 (0.68) | 1.32 (0.71 | 1.35 (1.02 | 1.24 (0.61) | 1.19 (0.38) |
| pt_min | 13.48 (2.74) | 13.60 (2.13) | 13.59 (2.70 | 13.35 (2.23 | 13.23 (2.09) | 13.43 (2.29) |
| pt_max | 15.46 (8.34) | 15.51 (6.46) | 15.32 (6.50 | 15.87 (10.43 | 14.55 (5.45) | 14.24 (3.79) |
| sodium_min | 137.61 (4.81) | 138.15 (4.36) | 137.71 (4.67 | 137.86 (4.71 | 138.11 (4.05) | 137.43 (5.12) |
| sodium_max | 141.10 (5.32) | 141.18 (4.69) | 141.06 (5.13 | 141.28 (5.13 | 140.64 (4.22) | 140.19 (4.30) |
| bun_min | 15.66 (10.74) | 15.87 (11.21) | 15.87 (11.12 | 15.65 (10.75 | 15.44 (10.38) | 15.00 (11.14) |
| bun_max | 18.57 (11.96) | 18.93 (12.36) | 18.80 (12.39 | 18.77 (11.96 | 18.25 (11.40) | 17.56 (12.13) |
| wbc_min | 9.99 (4.25) | 10.03 (4.45) | 9.90 (4.31 | 10.27 (4.31 | 9.54 (3.93) | 10.18 (3.91) |
| wbc_max | 13.21 (6.42) | 13.27 (6.30) | 13.19 (6.63 | 13.42 (5.77 | 12.45 (5.82) | 12.61 (5.09) |
| calcium_min | 8.19 (0.97) | 8.21 (0.98) | 8.17 (0.97 | 8.27 (0.95 | 8.35 (0.88) | 8.28 (0.83) |
| calcium_max | 8.77 (0.69) | 8.74 (0.77) | 8.76 (0.68 | 8.76 (0.79 | 8.80 (0.70) | 8.78 (0.68) |

Data are n (%) or mean (SD). Two-tailed Student’s t-tests or Mann-Whitney U-test for continuous variables, Chi-squared or Fisher’s exact test for categorical variables. *=P<0.05. SBP: systolic blood pressure; DBP: diastolic blood pressure; MBP: mean blood pressure; SpO2: oxygen saturation; PTT: partial thromboplastin time; INR: International Normalized Ratio; PT: prothrombin time; BUN: blood urea nitrogen; WBC: white blood cell.

**Supplemental Table 5.** Hyper-parameter space with different machine learning algorithms.

| Models | Hyper-parameter space |
| --- | --- |
| KNN | {'algorithm': ['auto', 'ball_tree', 'kd_tree', 'brute'], 'leaf_size': [1, 5, 10, 20, 30, 40, 50], 'n_neighbors': [1, 2, 3, 4, 5, 6, 7, 8, 9, 10, 11, 12, 13, 14, 15, 16, 17, 18, 19, 20, 21, 22, 23, 24, 25], 'p': [1, 2, 3, 4, 5], 'weights': ['uniform', 'distance']} |
| MLP | {'alpha': [0.1, 0.01, 0.001, 0.0001], 'hidden_layer_sizes':[(50,),(100,)], 'solver': ['sgd', 'adam'], 'activation':['tanh','relu'], 'learning_rate':['constant', 'adaptive']} |
| XGBoost | {'max_depth':[1, 2, 3, 5, 6, 7, 9, 12, 15, 17, 25], 'gamma':[ 0, 0.05 ,0.1,0.2, 0.3, 0.5, 0.7, 0.9, 1], 'subsample':[ 0.6, 0.7, 0.8, 0.9, 1], 'colsample_bytree':[0.6, 0.7, 0.8, 0.9, 1], 'learning_rate':[0.01, 0.015, 0.025, 0.05, 0.1], 'n_estimators':[100,200,300,400,500,600,700,800,900,1000]} |

Hyper-parameters not listed in the table were set to default values predefined in the scikit-learn module (version 1.0.2). KNN – k-nearest neighbor, MLP – multi-layer perceptron, XGBoost – extreme gradient boosting.

**Supplemental Table 6.** The International Classification of Diseases codes of the patients included in the study.

| Code Range | Description |  |
| --- | --- | --- |
| 80.000-80.049 | Closed fracture of vault of skull |  |
| 80.050-80.099 | Open fracture of vault of skull |  |
| 80.100-80.149 | Closed fracture of base of skull |  |
| 80.150-80.199 | Open fracture of base of skull |  |
| 80.300-80.349 | Other closed skull fracture |  |
| 80.350-80.399 | Other open skull fracture |  |
| 80.400-80.449 | Closed fractures involving skull or face with other bones |  |
| 80.450-80.499 | Open fractures involving skull or face with other bones |  |
| 85.00-85.09 | Concussion |  |
| 85.100-85.119 | Cortex (cerebral) contusion |  |
| 85.120-85.139 | Cortex (cerebral) laceration |  |
| 85.140-85.159 | Cerebellar or brain stem contusion |  |
| 85.160-85.179 | Cerebellar or brain stem laceration |  |
| 85.180-85,199 | Other and unspecified cerebral laceration and contusion |  |
| 85.200-85.219 | Subarachnoid hemorrhage following injury |  |
| 85.220-85.239 | Subdural hemorrhage following injury |  |
| 85.240-85.259 | Extradural hemorrhage following injury |  |
| 85.300-85.319 | Other and unspecified intracranial hemorrhage following injury |  |
| 85.400-85.419 | Intracranial injury of other and unspecified nature |  |

**Supplemental Table 7.** Electronic health records features overview.

| Categories | Features |
| --- | --- |
| Demographics | Age, gender, race, weight, height, and BMI |
| Intracranial injury types | Epidural hematoma, subdural hematoma, subarachnoid hemorrhage, intracerebral hemorrhage, cerebral contusion |
| Comorbidities | Metastatic solid tumor, aids, severe liver disease, cerebrovascular disease, renal disease, diabetes, malignant cancer, myocardial infarct, congestive heart failure, peripheral vascular disease, dementia, chronic pulmonary disease, rheumatic disease, peptic ulcer disease, mild liver disease, paraplegia |
| Vital signs | Heart rate, SBP, DBP, MBP, temperature, respiratory rate, SpO2 and urineoutput |
| Laboratory results | Anion gap, bicarbonate, creatinine, chloride, glucose, hematocrit, hemoglobin, platelet, potassium, ptt, inr, pt, sodium, bun, wbc and calcium |
| Medical treatment | Mechanical ventilation, RRT and vasopressor |
| Illness severity scoring systems | GCS, APS III and SOFA |

BMI: body mass index; SBP: systolic blood pressure; DBP: diastolic blood pressure; MBP: mean blood pressure; SpO2: oxygen saturation; partial thromboplastin time; INR: International Normalized Ratio; PT: prothrombin time; BUN: blood urea nitrogen; WBC: white blood cell; RRT: renal replacement therapy; GCS: Glasgow Coma Scale; APSIII: Acute Physiology Score III; SOFA: Sequential Organ Failure Assessment.

**Supplemental Table 8.** Positive predictive value (PPV) and negative predictive value (NPV) of the best performing eICU collaborative research database traumatic brain ianjury-cohort models for each clinical outcome. Best model is identified by an asterisk * in Table 2.

|  | Model type | PPV | NPV | Prevalence |
| --- | --- | --- | --- | --- |
| In-hospital mortality | Best model | 0.443 | 0.973 | 0.182 |
|  | APACHE IV | 0.500 | 0.967 | 0.182 |
|  | APS III | 0.557 | 0.949 | 0.182 |
|  | GCS | 0.072 | 0.544 | 0.182 |
|  | SOFA | 0.426 | 0.949 | 0.182 |
| Neurological outcome | Best model | 0.577 | 0.929 | 0.273 |
|  | APACHE IV | 0.516 | 0.939 | 0.273 |
|  | APS III | 0.543 | 0.924 | 0.273 |
|  | GCS | 0.132 | 0.421 | 0.273 |
|  | SOFA | 0.507 | 0.88 | 0.273 |
| prolonged length of ICU stay | Best model | 0.554 | 0.904 | 0.264 |
|  | APACHE IV | 0.405 | 0.838 | 0.264 |
|  | APS III | 0.449 | 0.859 | 0.264 |
|  | GCS | 0.128 | 0.449 | 0.264 |
|  | SOFA | 0.417 | 0.893 | 0.264 |

APACHE IV: Acute Physiology and Chronic Health Evaluation IV; APSIII: Acute Physiology Score III; GCS: Glasgow Coma Scale; SOFA: Sequential Organ Failure Assessment.

**Supplemental Table 9.** Model performance summary for all models for each clinical outcome and feature subset in training set.

|  | *Source* | *Model type* | *AUROC* | *Sensitivity* | *Specificity* | *F1 score* | *Accuracy* |
| --- | --- | --- | --- | --- | --- | --- | --- |
| *In-hospital mortality* | *EHR* | *KNN* | *0.744(0.676,* *0.813)* | *0.833(0.722, 0.926)* | *0.523(0.461, 0.585)* | *0.405(0.353, 0.462)* | *0.577(0.522, 0.631)* |
|  |  | *MLP* | *0.739(0.671,* *0.807)* | *0.741(0.611, 0.852)* | *0.663(0.605, 0.721)* | *0.442(0.388, 0.497)* | *0.676(0.625, 0.728)* |
|  |  | **XGBoost* | *0.884(0.833,* *0.935)* | *0.833(0.722, 0.926)* | *0.853(0.810, 0.895)* | *0.657(0.603, 0.708)* | *0.849(0.808, 0.888)* |
|  | *PTS* | *KNN* | *0.801(0.761,* *0.840)* | *0.752(0.674, 0.822)* | *0.713(0.675, 0.749)* | *0.495(0.458, 0.532)* | *0.720(0.686, 0.752)* |
|  |  | *MLP* | *0.885(0.853,* *0.918)* | *0.791(0.721, 0.860)* | *0.836(0.804, 0.865)* | *0.626(0.590, 0.662)* | *0.827(0.799, 0.854)* |
|  |  | **XGBoost* | *0.934(0.910,* *0.959)* | *0.786(0.714, 0.857)* | *0.934(0.912, 0.953)* | *0.756(0.724, 0.788)* | *0.906(0.885, 0.928)* |
|  | *EHR+PTS* | *KNN* | *0.892(0.853,* *0.931)* | *0.778(0.706, 0.849)* | *0.905(0.880, 0.928)* | *0.708(0.673, 0.741)* | *0.882(0.857, 0.905)* |
|  |  | *MLP* | *0.868(0.833,* *0.903)* | *0.778(0.706, 0.849)* | *0.842(0.812, 0.873)* | *0.628(0.592, 0.664)* | *0.830(0.801, 0.858)* |
|  |  | **XGBoost* | *0.943(0.923,* *0.964)* | *0.876(0.814, 0.930)* | *0.894(0.869, 0.919)* | *0.746(0.713, 0.778)* | *0.891(0.867, 0.914)* |
|  | *APACHE IV* | | *0.871(0.836,* *0.906)* | *0.762(0.683, 0.833)* | *0.847(0.815, 0.876)* | *0.630(0.592, 0.666)* | *0.831(0.802, 0.859)* |
|  | *APS III* | | *0.863(0.827,* *0.899)* | *0.762(0.683, 0.833)* | *0.834(0.802, 0.865)* | *0.615(0.579, 0.652)* | *0.820(0.790, 0.849)* |
|  | *GCS* | | *0.791(0.747,* *0.835)* | *0.742(0.664, 0.812)* | *0.779(0.744, 0.812)* | *0.544(0.507, 0.582)* | *0.772(0.741, 0.802)* |
|  | *SOFA* | | *0.798(0.756,* *0.840)* | *0.845(0.783, 0.907)* | *0.638(0.599, 0.676)* | *0.488(0.451, 0.525)* | *0.676(0.641, 0.710)* |
| *Neurological*  *Status* | *EHR* | *KNN* | *0.882(0.849,* *0.916)* | *0.708(0.619, 0.788)* | *0.855(0.816, 0.891)* | *0.664(0.619, 0.707)* | *0.818(0.782, 0.854)* |
|  |  | *MLP* | *0.763(0.710,* *0.816)* | *0.796(0.717, 0.867)* | *0.607(0.553, 0.659)* | *0.541(0.493, 0.588)* | *0.655(0.610, 0.698)* |
|  |  | **XGBoost* | *0.910(0.885,* *0.935)* | *0.881(0.835, 0.923)* | *0.793(0.758, 0.828)* | *0.725(0.692, 0.757)* | *0.817(0.789, 0.845)* |
|  | *PTS* | *KNN* | *0.798(0.762,* *0.834)* | *0.639(0.572, 0.706)* | *0.818(0.785, 0.851)* | *0.602(0.565, 0.637)* | *0.769(0.738, 0.800)* |
|  |  | *MLP* | *0.635(0.588,* *0.683)* | *0.562(0.490, 0.629)* | *0.689(0.648, 0.727)* | *0.470(0.433, 0.506)* | *0.654(0.619, 0.689)* |
|  |  | **XGBoost* | *0.885(0.858,* *0.913)* | *0.845(0.794, 0.897)* | *0.776(0.739, 0.810)* | *0.692(0.658, 0.726)* | *0.795(0.765, 0.824)* |
|  | *EHR+PTS* | *KNN* | *0.902(0.873,* *0.931)* | *0.758(0.682, 0.826)* | *0.879(0.846, 0.913)* | *0.727(0.686, 0.766)* | *0.846(0.814, 0.877)* |
|  |  | *MLP* | *0.805(0.768,* *0.842)* | *0.572(0.500, 0.639)* | *0.892(0.865, 0.917)* | *0.615(0.579, 0.651)* | *0.805(0.775, 0.833)* |
|  |  | **XGBoost* | *0.935(0.910,* *0.961)* | *0.894(0.832, 0.947)* | *0.876(0.840, 0.909)* | *0.792(0.755, 0.829)* | *0.881(0.849, 0.910)* |
|  | *APACHE IV* | | *0.853(0.819,* *0.887)* | *0.674(0.602, 0.740)* | *0.902(0.874, 0.927)* | *0.697(0.662, 0.731)* | *0.839(0.810, 0.866)* |
|  | *APS III* | | *0.847(0.813,* *0.880)* | *0.784(0.727, 0.840)* | *0.774(0.737, 0.809)* | *0.657(0.622, 0.691)* | *0.776(0.745, 0.807)* |
|  | *GCS* | | *0.810(0.775,* *0.845)* | *0.763(0.701, 0.820)* | *0.756(0.720, 0.793)* | *0.632(0.596, 0.668)* | *0.758(0.726, 0.789)* |
|  | *SOFA* | | *0.799(0.764,* *0.834)* | *0.835(0.784, 0.887)* | *0.658(0.617, 0.698)* | *0.608(0.572, 0.644)* | *0.706(0.672, 0.740)* |
| *prolonged length of*  *ICU stay* | *EHR* | *KNN* | *0.877(0.841,* *0.913)* | *0.832(0.769, 0.888)* | *0.848(0.813, 0.881)* | *0.728(0.692, 0.764)* | *0.844(0.814, 0.874)* |
|  |  | *MLP* | *0.718(0.668,* *0.767)* | *0.671(0.599, 0.743)* | *0.696(0.652, 0.739)* | *0.533(0.492, 0.574)* | *0.690(0.652, 0.728)* |
|  |  | **XGBoost* | *0.885(0.853,* *0.916)* | *0.803(0.737, 0.862)* | *0.835(0.800, 0.871)* | *0.709(0.672, 0.745)* | *0.827(0.795, 0.856)* |
|  | *PTS* | *KNN* | *0.801(0.761,* *0.840)* | *0.752(0.674, 0.822)* | *0.713(0.675, 0.749)* | *0.495(0.458, 0.532)* | *0.720(0.686, 0.752)* |
|  |  | *MLP* | *0.885(0.853,* *0.918)* | *0.791(0.721, 0.860)* | *0.836(0.804, 0.865)* | *0.626(0.590, 0.662)* | *0.827(0.799, 0.854)* |
|  |  | **XGBoost* | *0.918(0.894,* *0.942)* | *0.836(0.776, 0.895)* | *0.835(0.800, 0.871)* | *0.728(0.692, 0.764)* | *0.835(0.804, 0.865)* |
|  | *EHR+PTS* | **KNN* | *0.948(0.932,* *0.963)* | *0.952(0.916, 0.982)* | *0.771(0.733, 0.807)* | *0.721(0.687, 0.755)* | *0.816(0.786, 0.845)* |
|  |  | *MLP* | *0.817(0.776,* *0.859)* | *0.737(0.664, 0.803)* | *0.784(0.744, 0.821)* | *0.629(0.589, 0.669)* | *0.771(0.737, 0.806)* |
|  |  | *XGBoost* | *0.943(0.923,* *0.964)* | *0.876(0.814, 0.930)* | *0.894(0.869, 0.919)* | *0.746(0.713, 0.778)* | *0.891(0.867, 0.914)* |
|  | *APACHE IV* | | *0.633(0.577,* *0.689)* | *0.437(0.352, 0.521)* | *0.794(0.754, 0.832)* | *0.435(0.394, 0.478)* | *0.700(0.660, 0.739)* |
|  | *APS III* | | *0.663(0.607,* *0.719)* | *0.563(0.479, 0.648)* | *0.754(0.711, 0.797)* | *0.502(0.459, 0.545)* | *0.703(0.664, 0.743)* |
|  | *GCS* | | *0.698(0.649,* *0.747)* | *0.596(0.514, 0.678)* | *0.742(0.700, 0.783)* | *0.509(0.467, 0.550)* | *0.705(0.666, 0.742)* |
|  | *SOFA* | | *0.663(0.612,* *0.714)* | *0.717(0.645, 0.789)* | *0.555(0.508, 0.602)* | *0.484(0.444, 0.525)* | *0.598(0.558, 0.638)* |

EHR: electronic health record; PTS: physiological time series; APSIII: Acute Physiology Score III; GCS: Glasgow Coma Scale; SOFA: Sequential Organ Failure Assessment; KNN: K-Nearest Neighbor; MLP: Multi-Layer Perceptron; XGBoost: eXtreme Gradient Boosting; AUROC: Area Under the Receiver Operating Characteristic curve.

**Supplemental Table 10.** Model performance summary for medical information mart for intensive care external validation.

|  | *Source* | *Model type* | *AUROC* | *Sensitivity* | *Specificity* | *F1 score* | *Accuracy* |
| --- | --- | --- | --- | --- | --- | --- | --- |
| *In-hospital mortality* | *EHR* | *KNN* | *0.675(0.542,* *0.807)* | *0.724(0.552, 0.862)* | *0.615(0.462, 0.769)* | *0.646(0.529, 0.750)* | *0.662(0.544, 0.765)* |
|  |  | *MLP* | *0.728(0.608,* *0.847)* | *0.897(0.759, 1.000)* | *0.462(0.308, 0.615)* | *0.684(0.574, 0.794)* | *0.647(0.529, 0.765)* |
|  |  | **XGBoost* | *0.776(0.660,* *0.892)* | *0.690(0.517, 0.862)* | *0.769(0.641, 0.897)* | *0.690(0.574, 0.794)* | *0.735(0.632, 0.838)* |
|  | *PTS* | *KNN* | *0.730(0.609, 0.851)* | *0.828(0.690, 0.966)* | *0.564(0.410, 0.718)* | *0.686(0.574, 0.794)* | *0.676(0.559, 0.779)* |
|  |  | *MLP* | *0.769(0.649, 0.890)* | *0.655(0.483, 0.828)* | *0.846(0.718, 0.949)* | *0.704(0.588, 0.809)* | *0.765(0.662, 0.868)* |
|  |  | **XGBoost* | *0.756(0.627, 0.885)* | *0.793(0.621, 0.931)* | *0.795(0.667, 0.923)* | *0.767(0.662, 0.868)* | *0.794(0.691, 0.882)* |
|  | *EHR+PTS* | *KNN* | *0.690(0.560,* *0.819)* | *0.862(0.724, 0.966)* | *0.487(0.333, 0.641)* | *0.676(0.559, 0.779)* | *0.647(0.529, 0.765)* |
|  |  | *MLP* | *0.696(0.566,* *0.826)* | *0.655(0.483, 0.828)* | *0.744(0.590, 0.872)* | *0.655(0.544, 0.765)* | *0.706(0.588, 0.809)* |
|  |  | **XGBoost* | *0.779(0.666,* *0.892)* | *0.690(0.517, 0.862)* | *0.795(0.667, 0.923)* | *0.702(0.588, 0.809)* | *0.750(0.647, 0.853)* |
|  | *APS III* | | *0.649(0.515,* *0.784)* | *0.552(0.379, 0.724)* | *0.795(0.667, 0.923)* | *0.604(0.485, 0.721)* | *0.691(0.574, 0.794)* |
|  | *GCS* | | *0.581(0.441,* *0.722)* | *0.655(0.483, 0.828)* | *0.641(0.487, 0.795)* | *0.613(0.500, 0.721)* | *0.647(0.529, 0.765)* |
|  | *SOFA* | | *0.604(0.466,* *0.743)* | *0.379(0.207, 0.552)* | *0.821(0.692, 0.923)* | *0.468(0.353, 0.588)* | *0.632(0.515, 0.750)* |
| *Neurological*  *Status* | *EHR* | *KNN* | *0.671(0.541,* *0.801)* | *0.914(0.800, 1.000)* | *0.455(0.273, 0.636)* | *0.753(0.647, 0.853)* | *0.691(0.574, 0.794)* |
|  |  | *MLP* | *0.675(0.546,* *0.805)* | *0.800(0.657, 0.914)* | *0.515(0.333, 0.697)* | *0.709(0.603, 0.809)* | *0.662(0.544, 0.765)* |
|  |  | **XGBoost* | *0.758(0.642,* *0.875)* | *0.829(0.686, 0.943)* | *0.667(0.515, 0.818)* | *0.773(0.676, 0.868)* | *0.750(0.647, 0.853)* |
|  | *PTS* | *KNN* | *0.722(0.595,* *0.849)* | *0.771(0.629, 0.914)* | *0.667(0.515, 0.818)* | *0.740(0.632, 0.838)* | *0.721(0.618, 0.824)* |
|  |  | *MLP* | *0.635(0.500,* *0.769)* | *0.543(0.371, 0.714)* | *0.788(0.636, 0.909)* | *0.623(0.500, 0.735)* | *0.662(0.544, 0.765)* |
|  |  | **XGBoost* | *0.745(0.618,* *0.871)* | *0.714(0.571, 0.857)* | *0.879(0.758, 0.970)* | *0.781(0.676, 0.868)* | *0.794(0.691, 0.882)* |
|  | *EHR+PTS* | *KNN* | *0.725(0.601,* *0.848)* | *0.629(0.457, 0.771)* | *0.788(0.636, 0.909)* | *0.688(0.574, 0.794)* | *0.706(0.588, 0.809)* |
|  |  | *MLP* | *0.623(0.488,* *0.757)* | *0.857(0.743, 0.971)* | *0.394(0.242, 0.576)* | *0.706(0.588, 0.809)* | *0.632(0.515, 0.750)* |
|  |  | **XGBoost* | *0.780(0.666,* *0.894)* | *0.743(0.600, 0.886)* | *0.788(0.636, 0.909)* | *0.765(0.662, 0.868)* | *0.765(0.662, 0.868)* |
|  | *APS III* | | *0.570(0.431,* *0.710)* | *0.486(0.314, 0.657)* | *0.788(0.636, 0.909)* | *0.576(0.456, 0.691)* | *0.632(0.515, 0.750)* |
|  | *GCS* | | *0.570(0.434,* *0.705)* | *0.629(0.457, 0.771)* | *0.667(0.515, 0.818)* | *0.647(0.529, 0.765)* | *0.647(0.529, 0.765)* |
|  | *SOFA* | | *0.552(0.412,* *0.691)* | *0.343(0.200, 0.514)* | *0.818(0.667, 0.939)* | *0.453(0.338, 0.574)* | *0.574(0.456, 0.691)* |
| *prolonged length of*  *ICU stay* | *EHR* | *KNN* | *0.562(0.375,* *0.749)* | *1.000(1.000, 1.000)* | *0.379(0.207, 0.552)* | *0.526(0.359, 0.692)* | *0.538(0.385, 0.692)* |
|  |  | *MLP* | *0.721(0.542,* *0.899)* | *0.800(0.500, 1.000)* | *0.690(0.517, 0.862)* | *0.593(.436, 0.744)* | *0.718(0.564, 0.846)* |
|  |  | **XGBoost* | *0.755(0.601,* *0.910)* | *0.900(0.700, 1.000)* | *0.690(0.517, 0.862)* | *0.643(0.487, 0.795)* | *0.744(0.590, 0.872)* |
|  | *PTS* | *KNN* | *0.659(0.448,* *0.869)* | *0.600(0.300, 0.900)* | *0.759(0.586, 0.897)* | *0.522(0.359, 0.667)* | *0.718(0.564, 0.846)* |
|  |  | *MLP* | *0.666(0.435,* *0.896)* | *0.500(0.200, 0.800)* | *0.897(0.759, 1.000)* | *0.556(0.410, 0.718)* | *0.795(0.667, 0.923)* |
|  |  | **XGBoost* | *0.683(0.505,* *0.860)* | *1.000(1.000, 1.000)* | *0.379(0.207, 0.552)* | *0.526(0.359, 0.692)* | *0.538(0.385, 0.692)* |
|  | *EHR+PTS* | *KNN* | *0.569(0.367,* *0.771)* | *0.200(0.000, 0.500)* | *0.552(0.379, 0.724)* | *0.160(0.051, 0.282)* | *0.462(0.308, 0.615)* |
|  |  | *MLP* | *0.655(0.486,* *0.824)* | *1.000(1.000, 1.000)* | *0.483(0.310, 0.655)* | *0.571(0.410, 0.718)* | *0.615(0.462, 0.769)* |
|  |  | **XGBoost* | *0.769(0.618,* *0.920)* | *1.000(1.000, 1.000)* | *0.552(0.379, 0.724)* | *0.606(0.462, 0.744)* | *0.667(0.513, 0.821)* |
|  | *APS III* | | *0.710(0.527,* *0.894)* | *0.600(0.300, 0.900)* | *0.724(0.552, 0.862)* | *0.500(0.333, 0.667)* | *0.692(0.538, 0.821)* |
|  | *GCS* | | *0.533(0.307,* *0.758)* | *0.200(0.000, 0.500)* | *0.931(0.828, 1.000)* | *0.286(0.154, 0.436)* | *0.744(0.590, 0.872)* |
|  | *SOFA* | | *0.690(0.473,* *0.906)* | *0.500(0.200, 0.800)* | *0.931(0.828, 1.000)* | *0.588(0.436, 0.744)* | *0.821(0.692, 0.923)* |

EHR: electronic health record; PTS: physiological time series; APSIII: Acute Physiology Score III; GCS: Glasgow Coma Scale; SOFA: Sequential Organ Failure Assessment; KNN: K-Nearest Neighbor; MLP: Multi-Layer Perceptron; XGBoost: eXtreme Gradient Boosting; AUROC: Area Under the Receiver Operating Characteristic curve.

**Supplemental Figures**


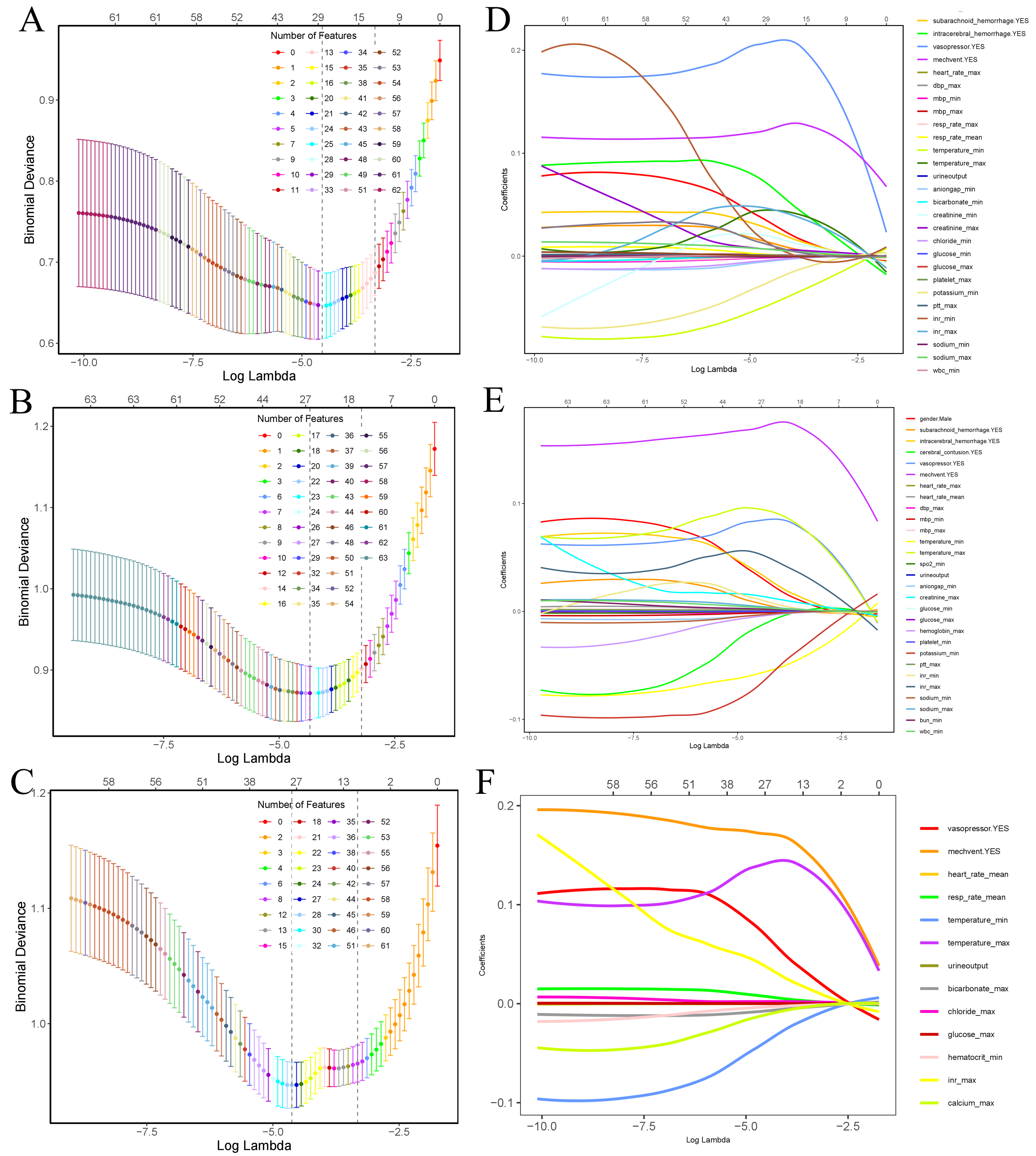


**Supplemental Figure 1**. Selection of indicators with prognostic electronic health record features in the training set. (A-C) Selection of optimal parameter (lambda) in least absolute shrinkage and selection operator model, the lambda value with the best predictive performance during cross-validation is considered as the optimal lambda value of outcomes in-hospital mortality (A), neurological status at hospital discharge (B), and prolonged length of ICU stay (C), respectively. (D-F) Generate coefficient profile plot for electronic health record indicators with prognostic information based on the sequence of logarithmic (lambda) values of outcomes in-hospital mortality (D), neurological status at hospital discharge (E), and prolonged length of ICU stay (F), respectively.


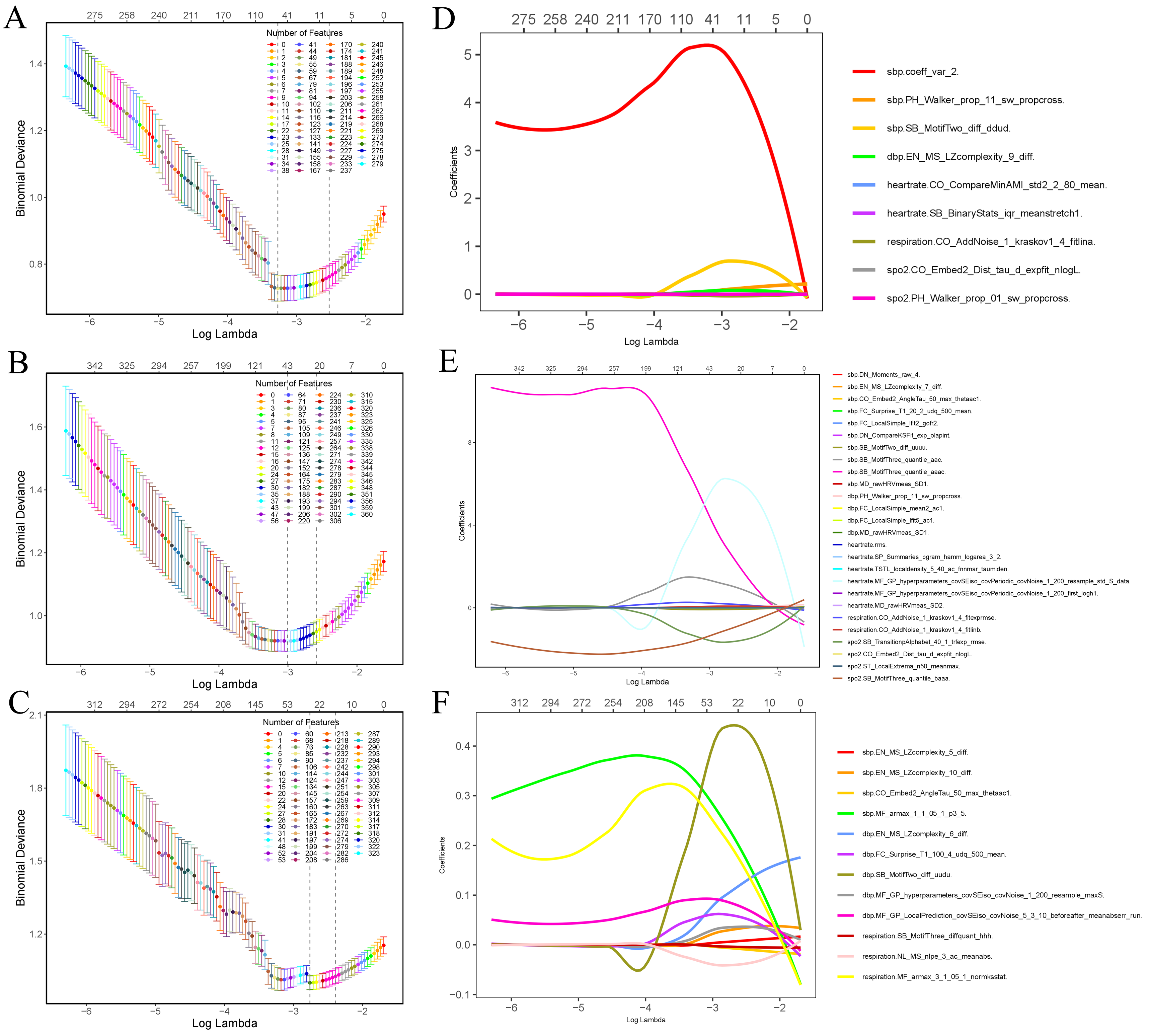


**Supplemental Figure 2**. Selection of indicators with prognostic highly comparative time-series analysis physiological time series derived features in the training set. (A-C) Selection of optimal parameter (lambda) in least absolute shrinkage and selection operator model, the lambda value with the best predictive performance during cross-validation is considered as the optimal lambda value of outcomes in-hospital mortality (A), neurological status at hospital discharge (B), and prolonged length of ICU stay (C), respectively. (D-F) Generate coefficient profile plot for highly comparative time-series analysis physiological time series derived features with prognostic information based on the sequence of logarithmic (lambda) values of outcomes in-hospital mortality (D), neurological status at hospital discharge (E), and prolonged length of ICU stay (F), respectively.


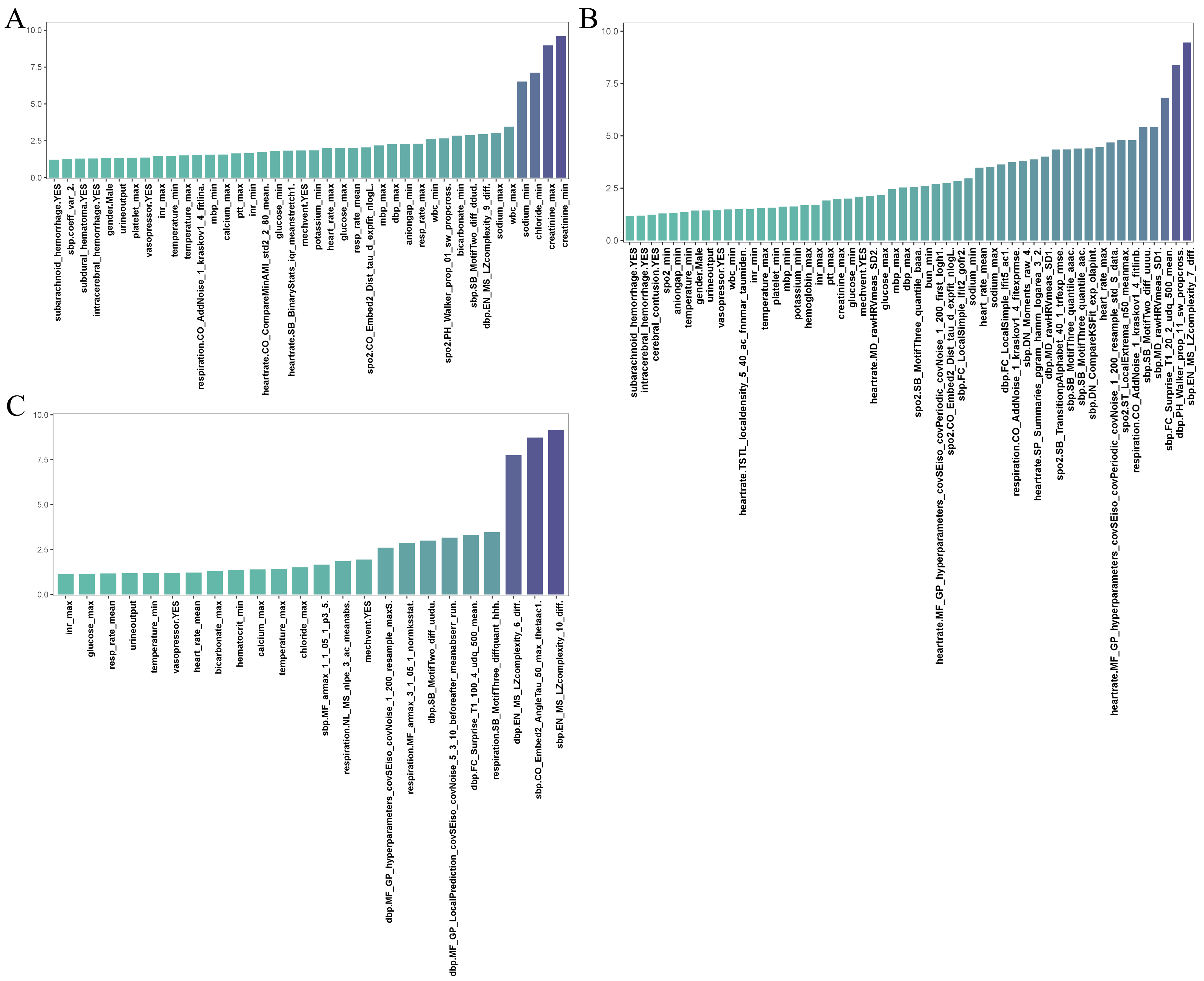


**Supplemental Figure 3**. The variance inflation factor results of the multicollinearity analysis of the electronic health record and highly comparative time-series analysis physiological time series derived features of outcomes in-hospital mortality (A), neurological status at hospital discharge (B), and prolonged length of ICU stay (C), respectively, in the training set.


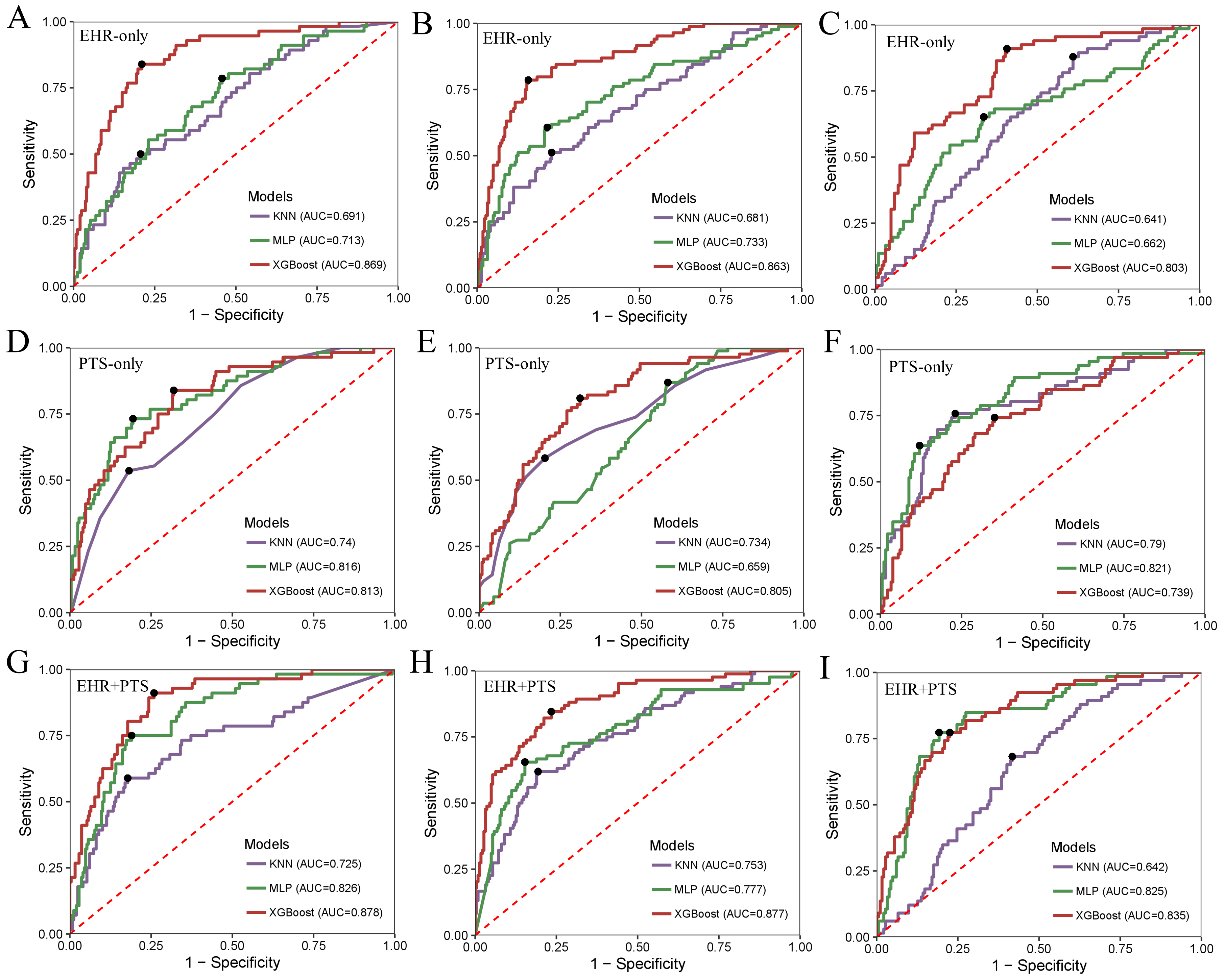


**Supplemental Figure 4**. Evaluation of predictive models for three clinical outcomes using Receiver Operating Characteristic curves in the testing set. (A, D, G) Predictive results of in-hospital mortality; (B, E, H) Predictive results of neurological status at hospital discharge; (C, F, I) Predictive results of prolonged length of ICU stay.


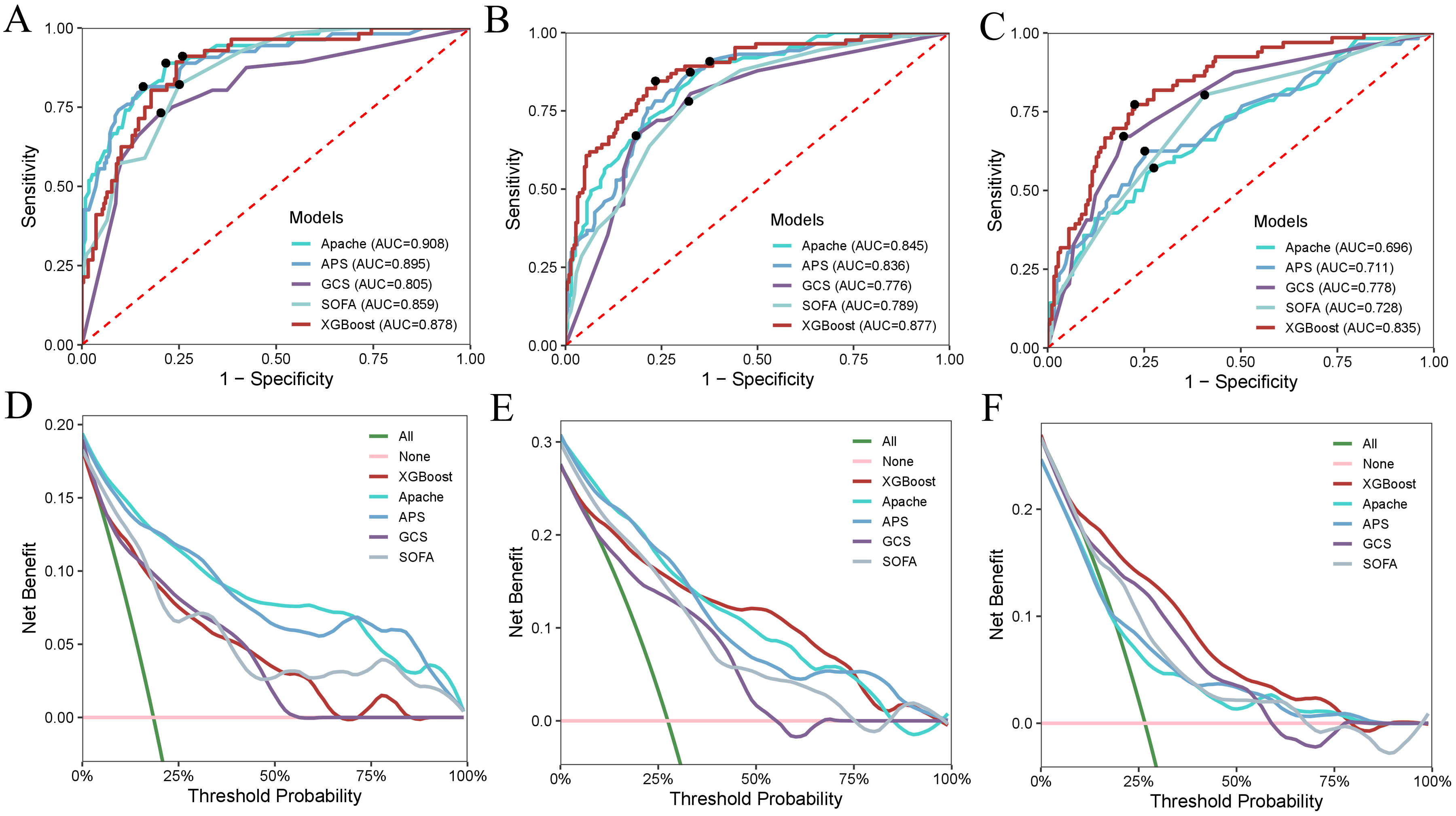


**Supplemental Figure 5**. Evaluation of the optimal machine learning model based on the combination of Electronic Health Record and PTS datasets compared to traditional scoring systems in the testing set. Comparison of Receiver Operating Characteristic curves separately for in-hospital mortality(A), neurological status at hospital discharge(B), and prolonged length of ICU stay(C). Comparison of Decision Curve Analysis curves separately for in-hospital mortality(D), neurological status at hospital discharge(E), and prolonged length of ICU stay(F).


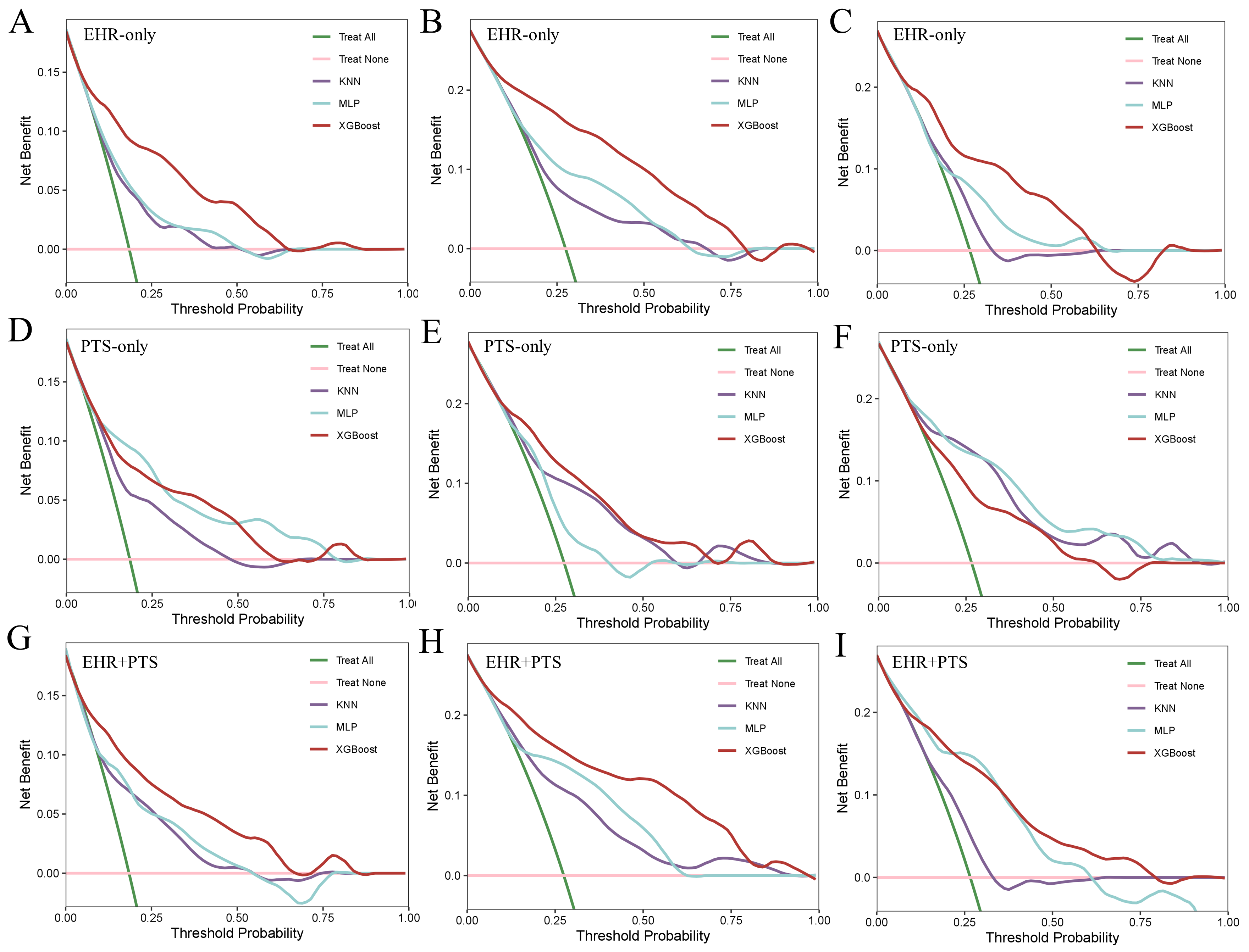


**Supplemental Figure 6**. Evaluation of predictive models using decision curve analysis curves of outcomes in-hospital mortality (A, D, G), neurological status at hospital discharge (B, E, H), and prolonged length of ICU stay (C, F, I), respectively, in the testing set.


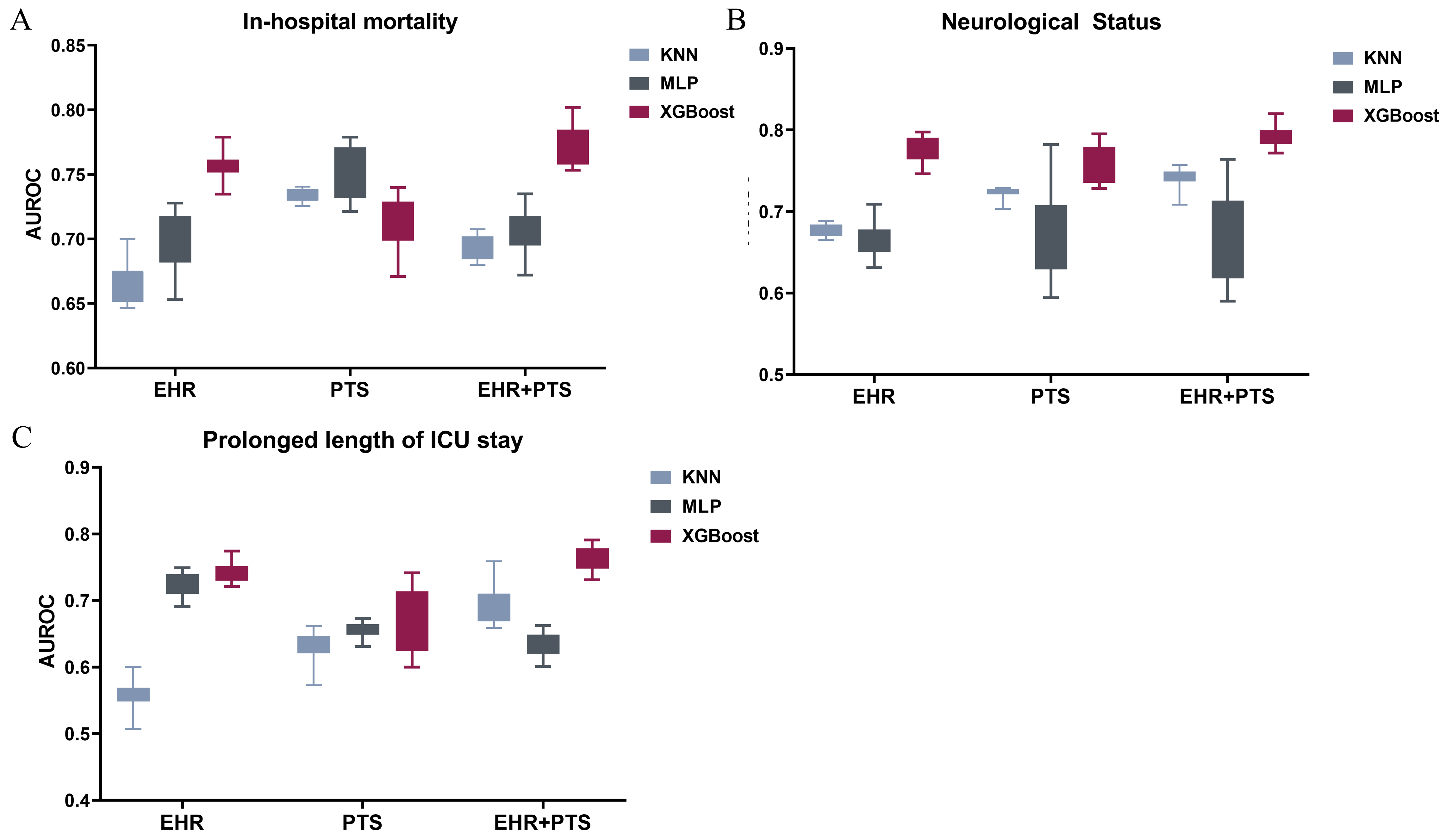


**Supplemental Figure 7.** Boxplots illustrating the results of 10-fold cross-validation with 50 repetitions on the entire dataset to validate and evaluate the external validation set outcomes. (A) Mortality as the outcome. (B) Neurological status at hospital discharge as the outcome. (C) Prolonged length of ICU stay as the outcome.


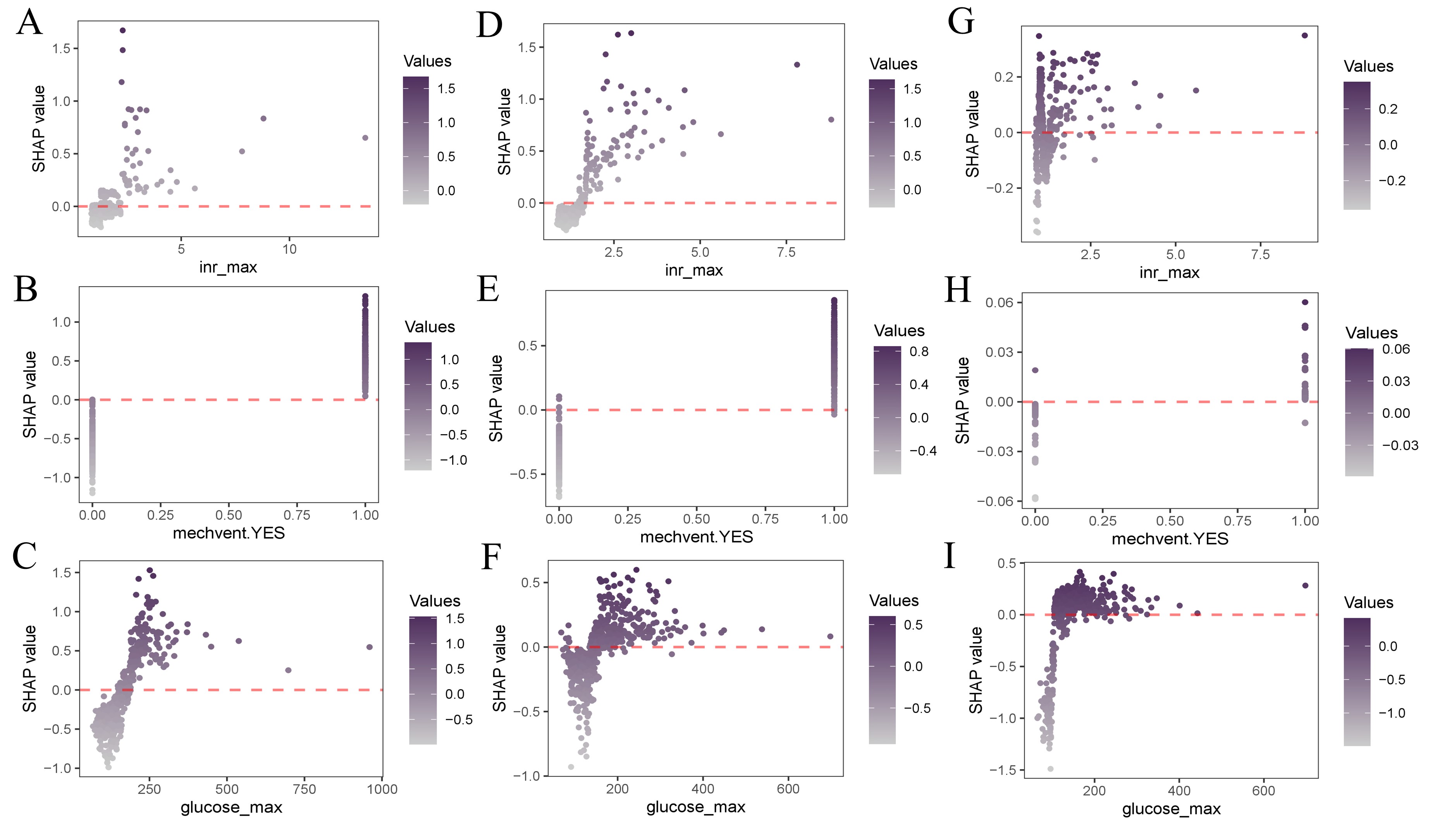


**Supplemental Figure 8.** Dependence plot based on shapley additive explanations values shows the influence of a single feature on the XGBoost-based prediction model output. Shapley additive explanations values less than zero were considered as a threshold and were regarded as the protective factors. (A-I) Shapley additive explanations values of inr_max, mechvent.YES, glucose_max of outcomes in-hospital mortality (A-C), neurological status at hospital discharge (D-F), and prolonged length of ICU stay (G-I).


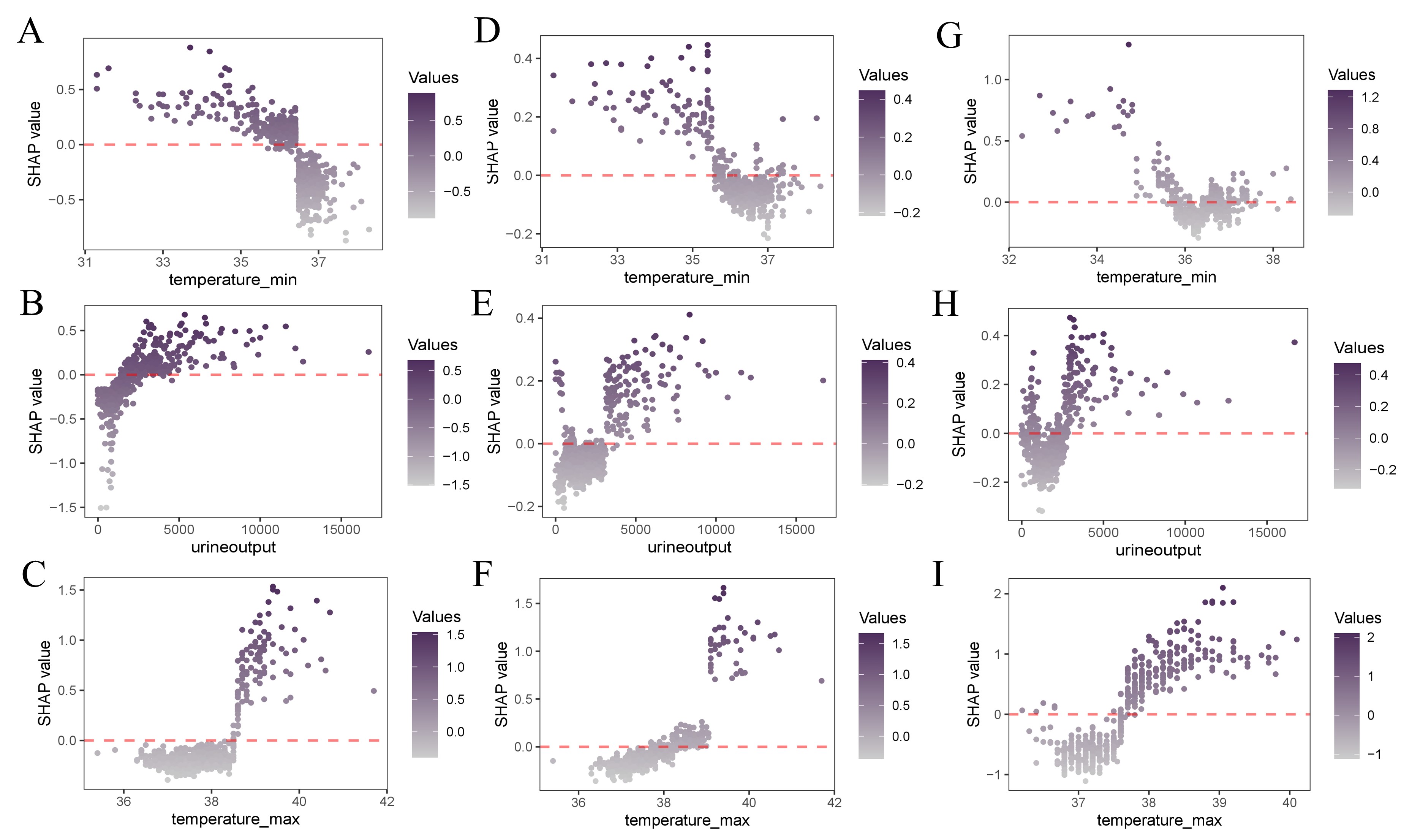


**Supplemental Figure 9.** Dependence plot based on shapley additive explanations values shows the influence of a single feature on the XGBoost-based prediction model output. Shapley additive explanations values less than zero were considered as a threshold and were regarded as the protective factors. (A-I) Shapley additive explanations values of temperature_min, urineoutput, and temperature_max of outcomes in-hospital mortality (A-C), neurological status at hospital discharge (D-F), and prolonged length of ICU stay (G-I).


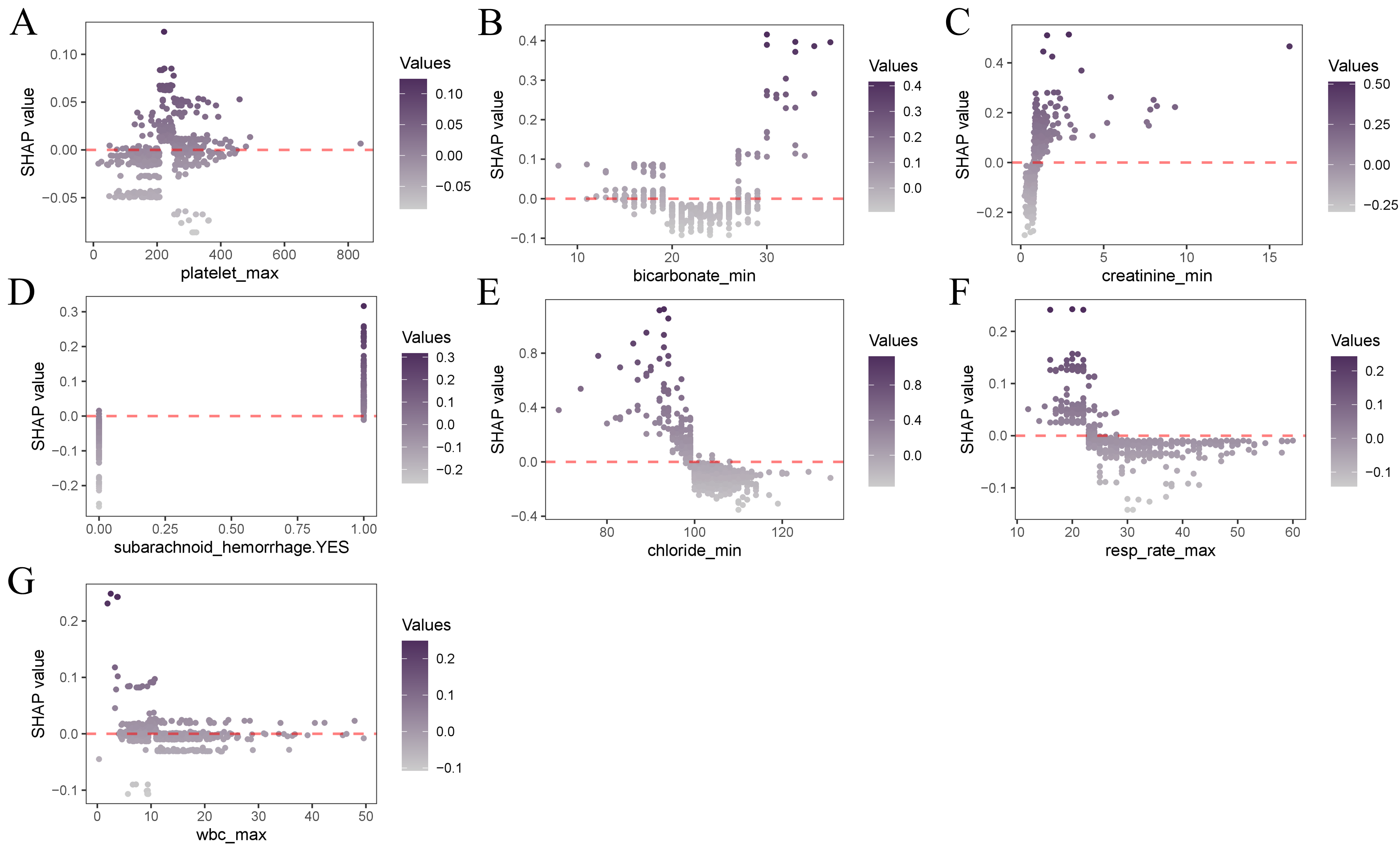


**Supplemental Figure 10**. The shapley additive explanations scores for XGBoost-based unique electronic health record predictor displayed as a bee diagram, with in-hospital mortality as the outcome. (A-G) Shapley additive explanations values of platelet_max, bicarbonate_min, creatinine_min, subdural_hematoma.YES, chloride_min, resp_rate_max, wbc_max.


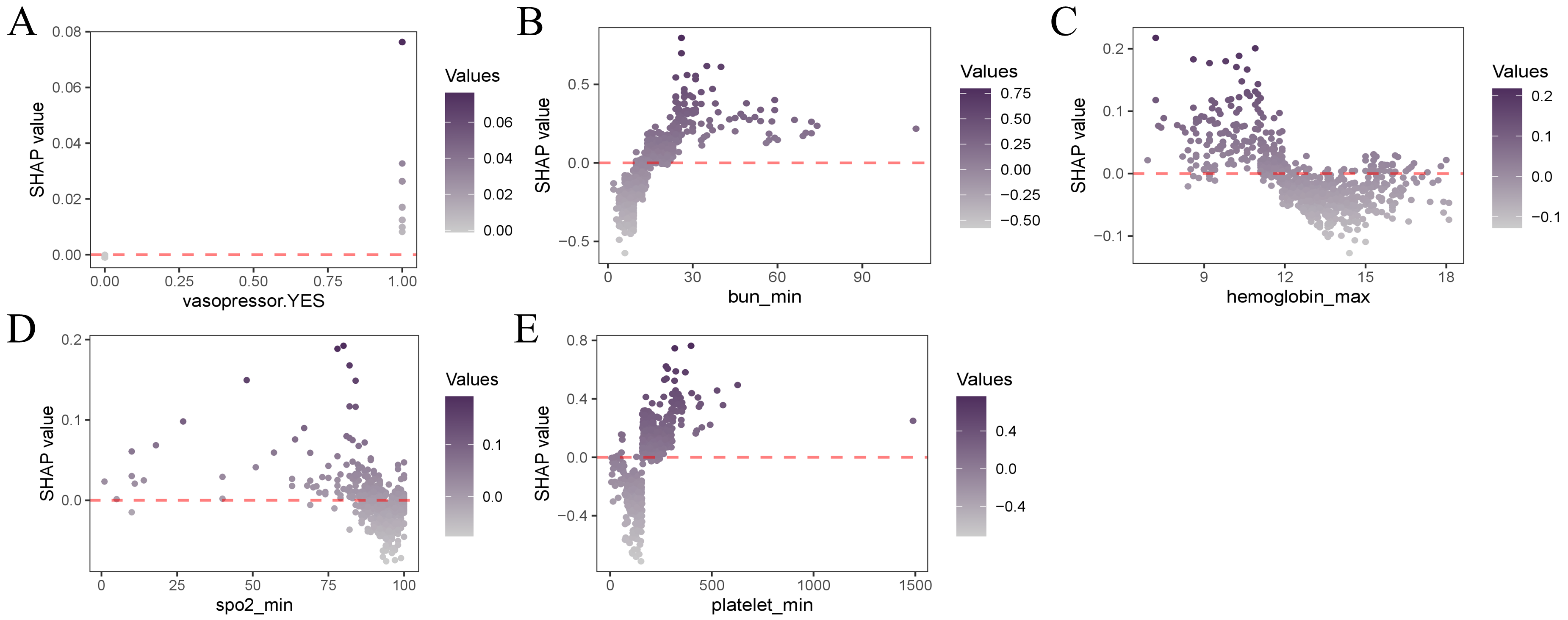


**Supplemental Figure 11**. The shapley additive explanations scores for XGBoost-based unique electronic health record predictor displayed as a bee diagram, with neurological status at hospital discharge as the outcome. (A-E) Shapley additive explanations values of vasopressor.YES, bun_min, hemoglobin_max, spo2_min, platelet_min.


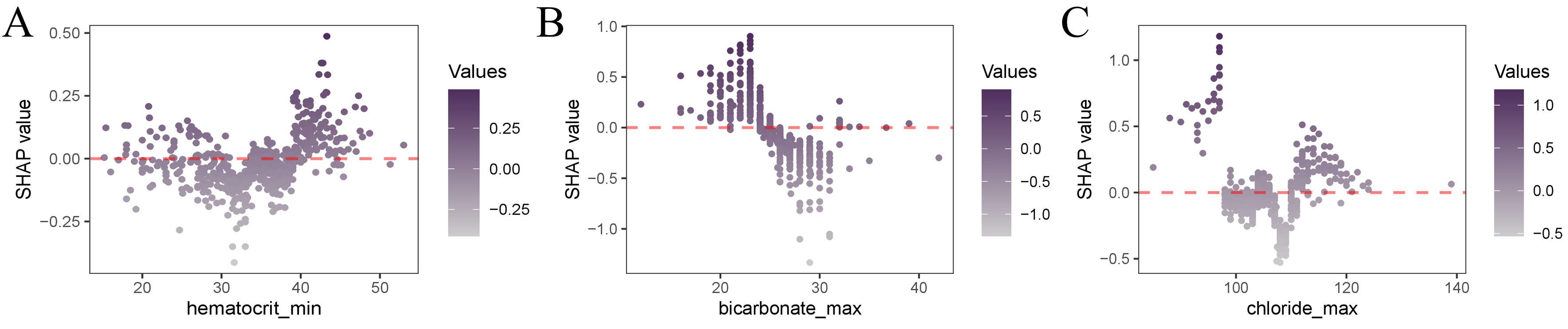


**Supplemental Figure 12**. The shapley additive explanations scores for XGBoost-based unique electronic health record predictor displayed as a bee diagram, with prolonged length of ICU stay as the outcome. (A-C) Shapley additive explanations values of hematocrit_min, bicarbonate_max, chloride_max.
